# Differential Expression of Insulin-Like Growth Factor 1 and Wnt Family Member 4 Correlates With Functional Heterogeneity of Human Dermal Fibroblasts

**DOI:** 10.3389/fcell.2021.628039

**Published:** 2021-04-06

**Authors:** Oliver J. Culley, Blaise Louis, Christina Philippeos, Bénédicte Oulès, Matthieu Tihy, Joe M. Segal, Della Hyliands, Gail Jenkins, Ranjit K. Bhogal, Richard C. Siow, Fiona M. Watt

**Affiliations:** ^1^Centre for Stem Cells and Regenerative Medicine, King’s College London, Guy’s Hospital, London, United Kingdom; ^2^Unilever R&D Colworth, Colworth Science Park, Bedford, United Kingdom; ^3^Cardiovascular Division, Faculty of Life Sciences and Medicine, King’s College London, London, United Kingdom

**Keywords:** skin, fibroblasts, signalling, epidermis, cell culture, IGF1, WNT4, α-SMA

## Abstract

Although human dermis contains distinct fibroblast subpopulations, the functional heterogeneity of fibroblast lines from different donors is under-appreciated. We identified one commercially sourced fibroblast line (c64a) that failed to express α-smooth muscle actin (α-SMA), a marker linked to fibroblast contractility, even when treated with transforming growth factor-β1 (TGF-β1). Gene expression profiling identified insulin-like growth factor 1 (IGF1) as being expressed more highly, and Asporin (ASPN) and Wnt family member 4 (WNT4) expressed at lower levels, in c64a fibroblasts compared to three fibroblast lines that had been generated in-house, independent of TGF-β1 treatment. TGF-β1 increased expression of C-X-C motif chemokine ligand 1 (CXCL1) in c64a cells to a greater extent than in the other lines. The c64a gene expression profile did not correspond to any dermal fibroblast subpopulation identified by single-cell RNAseq of freshly isolated human skin cells. In skin reconstitution assays, c64a fibroblasts did not support epidermal stratification as effectively as other lines tested. In fibroblast lines generated in-house, shRNA-mediated knockdown of IGF1 increased α-SMA expression without affecting epidermal stratification. Conversely, WNT4 knockdown had no consistent effect on α-SMA expression, but increased the ability of fibroblasts to support epidermal stratification. Thus, by comparing the properties of different lines of cultured dermal fibroblasts, we have identified IGF1 and WNT4 as candidate mediators of two distinct dermal functions: myofibroblast formation and epidermal maintenance.

## Introduction

Mammalian dermis contains fibroblasts that arise from different developmental lineages and have different functions (Sorrell and Caplan, 2004; Driskell et al., 2013; Rinkevich et al., 2015; Lichtenberger et al., 2016; Lynch and Watt, 2018; Philippeos et al., 2018; Shook et al., 2018; Tabib et al., 2018; Guerrero-Juarez et al., 2019; Korosec et al., 2019). In mouse dorsal skin, there are at least three distinct lineages, giving rise to the Sox2+ dermal papillae of awl/auchene hair follicles and the cells of the upper and lower dermis (Driskell et al., 2013; Rognoni and Watt, 2018). The upper lineage gives rise to Sox2- dermal papillae, arrector pili muscles, and papillary fibroblasts, while the lower lineage forms the reticular dermis and dermal adipocytes. The upper dermal lineage is required for epidermal maintenance and hair follicle reconstitution, while the lower dermal lineage is the first to repopulate full thickness wounds and gives rise to cells expressing α-smooth muscle actin (α-SMA).

Attempts to define human skin fibroblast subpopulations based on mouse–human conservation of specific cell surface markers have met with limited success, despite conservation of specific gene signatures characteristic of Wnt signalling, cytokine signalling, and extracellular matrix (ECM) remodelling (Philippeos et al., 2018). Single-cell RNAseq of fibroblasts isolated directly from human dermis has yielded markers of different fibroblasts (Philippeos et al., 2018; Tabib et al., 2018; Guerrero-Juarez et al., 2019; Solé-Boldo et al., 2020; Reynolds et al., 2021). While some of the cell surface markers that distinguish fibroblasts *in vivo* can be used to select subpopulations by flow cytometry, the expression of the markers is often rapidly decreased in culture. Nevertheless, in both mouse and human, there is evidence that the distinct functionality of different fibroblast subsets can be preserved following expansion in culture (Driskell et al., 2011, 2012, 2013; Fujiwara et al., 2011; Philippeos et al., 2018).

Unfractionated dermal fibroblasts, expanded in culture, have been injected into patients for potential therapeutic applications, including wound healing, scar repair, and alleviation of Recessive Dystrophic Epidermolysis Bullosa (Petrof et al., 2013; Rashidghamat and McGrath, 2017; Bajouri et al., 2020). For some indications, injection of specific fibroblast subsets could be beneficial (Lynch and Watt, 2018): for example, papillary fibroblasts would be expected to support epidermal maintenance, while reticular fibroblasts would maintain the hypodermis. However, the properties of both normal and cancer cells change over time in culture, with selective outgrowth of cells with dominant characteristics (Hughes et al., 2007; Ben-David et al., 2019). This led us to explore whether commercial fibroblast lines cultured from different donors might differ in functionality and, if so, whether underlying differences in gene expression might account for functional differences.

## Materials and Methods

### Human Tissue

Full thickness surgical waste skin from healthy adult volunteers was obtained with appropriate ethical approval (REC 14/NS/1073) from the Department of Plastic and Reconstructive Surgery, St George’s Hospital, London.

### Cell Culture

Normal human dermal fibroblasts (NHDF) were either isolated directly from surgical waste skin as described previously (Philippeos et al., 2018) or purchased, cryopreserved at passage 2, from PromoCell (C-12302). Supplementary Table 1 lists all of the fibroblasts studied. The selection of particular in-house lines for specific experiments was based on two criteria: availability of the lines at the time when the commercial cells were being characterised; and matching commercial and in-house lines in terms of passage number and age of donor.

The PromoCell lines were generated from fibroblasts that had been flow sorted for CD90 expression prior to plating. The PromoCell lines were from donors aged 19y (NHDF-c19, Lot: 4032503.1), 24y (NHDF-c24, Lot: 4081903.2), 64y (NHDF-c64a, Lot: 4012203.1), or 64y (NHDF-c64b, Lot: 3102301.3). Human keratinocytes (Km strain) from neonatal foreskin were isolated and cultured on a 3T3 J2 feeder layer as described previously (Philippeos et al., 2018). The culture conditions are described in Supplementary Materials and Methods.

STR profiles (Supplementary Table 2) were generated for the commercial fibroblast lines and the in-house lines used in the microarrays using PowerPlex assays (Promega; performed by Source BioScience, Nottingham, United Kingdom). The following loci were tested: AMEL, CSF1PO, D13S317, D16S539, D18S51, D21S11, D3S1358, D5S818, D7S820, D8S1179, FGA, Penta D, Penta E, TH01, TPOX, and vWA.

### RNA Extraction and qPCR

RNA was isolated using the RNeasy mini kit (Qiagen). The QuantiTect reverse transcription kit (Qiagen) was used to generate cDNA. cDNA was loaded into quadruplicate wells of a 384-well PCR plate (Bio-Rad). Reactions were run using TaqMan fast universal PCR master mix and TaqMan qPCR gene expression probes. Results are presented as quantitation cycle (Cq) values normalised using reference gene Cq values and displayed as ΔCq or ΔΔCq expression (Livak and Schmittgen, 2001). The TaqMan assays are listed in Supplementary Table 3.

### Agilent Gene Expression Microarray and Fluidigm 96:96 TaqMan qPCR

Normal human dermal fibroblasts were seeded into six-well microplates at 40,000 cells/well. NHDF-F22Br (female 22y breast skin), M50F (male 50y face skin), F60Br (female 60y breast skin), and PromoCell NHDF-c64a were cultured in complete DMEM for 4 days. NHDF were equilibrated in low (1%) serum DMEM for 24 h and treated with or without transforming growth factor-β1 (TGF-β1) (10 ng/ml) in low serum DMEM for a further 12 or 24 h. Cells were lysed and RNA was extracted using the RNeasy mini kit protocol. RNA concentration and integrity were measured using a Bioanalyser 2100 (Agilent) and RNA 6000 Nano kit (Agilent) according to the manufacturer’s instructions. The one-colour Quick-Amp labelling kit (Agilent) was used to derive cyanine 3-cytidine triphosphate (Cy3-CTP) labelled cRNA, and the spike-in kit (Agilent) was used to monitor sample amplification and labelling efficiency. RNA was converted to cDNA using the cDNA mix kit (Quick-Amp). See Supplementary Materials and Methods for hybridisation and analysis protocols.

### Computational Analysis

The single-cell RNA sequencing dataset from Tabib et al. (2018) was reanalysed using R^1^ and the “simpleSingleCell” package (Lun et al., 2016).

### shRNA-Based Gene Knockdown

One control shRNA plasmid (Sigma–Aldrich, SHC016-1EA), and four target shRNA plasmids (Sigma–Aldrich) were commercially sourced and purified using the Nucleobond Xtra midi plus EF kit (Thermo Scientific). Plasmids were introduced into HEK293 cells using jetPRIME transfection reagent (Polyplus Transfection). After 48 h, the medium was passed through a 0.45-μm filter (Corning), mixed with 1X Lenti-X concentrator (Clontech Laboratories), and stored at 4°C for 48 h. NHDF were transduced in polybrene (1:1000; Sigma–Aldrich) for 16 h, cultured in complete DMEM (DMEM supplemented with 10% FBS, 450 μg/ml L-glutamine, 100 U/ml penicillin, and 100 μg/ml streptomycin) for 8 h and then selected in 1 μg/ml puromycin (Thermo Scientific) for 3 days.

### Skin Reconstitution on De-Epidermised Dermis (DED)

The epidermis was removed from pieces of surgical waste skin and discarded. The dermis was subjected to repeated freeze-thaw cycles followed by gamma irradiation, as described previously (Philippeos et al., 2018). De-epidermised dermis (DED) was placed in six-well microplate Millicell hanging inserts (Millipore). 5 × 10^5^ NHDF were resuspended in 30 μl complete DMEM and injected into the upper dermis using a 0.3 ml insulin syringe and needle (Becton Dickinson). NHDF-containing DED was cultured submerged in 3 ml/well complete DMEM for 24 h. 1 × 10^6^ keratinocytes (strain Km) were resuspended in 30 μl complete FAD, seeded onto the DED, and cultured in 2 ml/well complete FAD above the air–liquid interface for 2 weeks. The histological analysis is described in Supplementary Materials and Methods.

### Data Availability

Datasets related to this article can be found in the Gene Expression Omnibus^2^, hosted by National Center for Biotechnology Information, under accession number: GSE140962.

## Results

### Identification of a Human Fibroblast Line That Does Not Express α-SMA in Culture

Four strains of commercially sourced (PromoCell) adult, NHDF were compared: c19, c24, c64a, and c64b (numbers refer to age of each donor). These cells had been isolated based on expression of the pan-fibroblast marker cluster of differentiation (CD)90 (THY1; Hagood et al., 2005; Hu and Barker, 2019) and were studied between passages 6 and 10. Cells from this commercial supplier have been injected into patients and evaluated therapeutically (Petrof et al., 2013).

**FIGURE 1:**
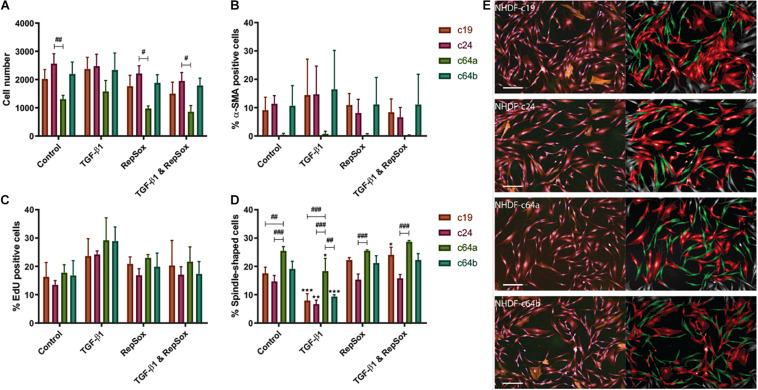
NHDF phenotypes. NHDF lines were untreated, treated with TGF-β1 (10 ng/ml) and/or RepSox (25 μM) in DMEM containing 1% FBS and assayed for cell number **(A)**, differentiation (% α-SMA positive, **B**), proliferation (% EdU positive, **C**), and shape (% spindle-shaped, **D**). Fluorescence intensity was thresholded on the maximum signal in unlabelled NHDF **(B,C)**. Error bars represent SD of mean values in triplicate wells of three 96-well microplates (*n* = 3). Two-way ANOVA comparing TGF-β1/RepSox treated versus own control NHDF (*), or c64a and different cell lines under the same condition (#) **(A–D)** (Tukey’s multiple comparisons test; ^#^*p* < 0.05, **,^##^*p* < 0.01, ***,^###^*p* < 0.001). **(E)** Representative images of PromoCell NHDF (c19, c24, c64a, and c64b) cultured in low (1%) serum DMEM. (Left) Input image with NucBlue (blue) EdU (green), α-SMA (orange), and CellMask (red). (Right) Spindle-shaped (green) and non-spindle-shaped (red) cells. Cells on the image border (grey) were excluded from analysis. Scale bar: 200 μm.

We used a high content imaging system (Operetta, Perkin Elmer) to phenotype the lines (Supplementary Figure 1). Cells were cultured under standard conditions [DMEM with 10% fetal bovine serum (FBS)], followed by low (1%) serum DMEM (24 h serum-starved) alone or in combination with TGF-β1. The rationale for the switch to low serum was that FBS not only contains high levels of latent TGF-β1 (Oida and Weiner, 2010) but contains a range of mitogens that could mask the stimulatory effect of TGF-β1 on proliferation. Cells were also treated with TGF-β1 together with the inhibitor RepSox, which targets ALK5 and thus blocks the Activin/BMP/TGF-β pathway Ichida et al., 2009. Figure 1 shows data from three biological replicates; each individual experiment from Figure 1 is shown in Supplementary Figures 2A–L. The statistical analysis in Figure 1 compares each cell line individually to c64a under the same conditions (hash signs) and control (untreated) versus treatment for each line (asterisks). One cell line, c64a, showed reduced cell number under control conditions or when treated with RepSox or RepSox and TGF-β1 (Figure 1A). We speculate that this reflects reduced cell adhesiveness on plating compared to the other lines.

Consistent with earlier studies (Clark et al., 1997; Liu et al., 2012), TGF-β1 treatment resulted in a modest increase in EdU incorporation in all cell lines, which was not statistically significant when biological replicates were combined (Figure 1C), but was significant in the case of at least one cell line in each individual experiment (Supplementary Figures 2A–L). TGF-β1 treatment also led to a reduction in spindle-shaped cells (Figure 1D). Fibroblasts tend to have a spindle/elongated morphology whereas myofibroblasts are more highly spread and round (Hinz et al., 2001). We trained the high content imaging software to determine “spindle” vs “round” populations, based on: morphology cell area (μm^2^); morphology cell roundness; and morphology cell ratio width to length. The analysis pipeline is presented in Supplementary Materials and Methods.

Three of the lines contained 10–20% α-SMA+ cells, regardless of culture conditions (Figure 1B). In contrast, the c64a line did not contain any α-SMA+ cells (Figure 1B) and had more spindle-shaped shaped cells than the other cell lines following TGF-β1 treatment (Figure 1D and Supplementary Figure 2). α-SMA is expressed by myofibroblasts, which are associated with cell and ECM contractility during normal wound healing and abnormal collagen deposition in scarring and fibrosis (Grinnell, 1994; Hinz et al., 2001; Tomasek et al., 2002; Klingberg et al., 2014; Lynch and Watt, 2018). There was a trend towards an increase in α-SMA+ cells on treatment with TGF-β1, except in the case of c64a cells (Figure 1B and Supplementary Figure 2). Figure 1E illustrates the morphological appearance of the four cell lines.

Supplementary Figures 2M–P show a comparison of c24 and c64a cells—two of the commercial lines—with two non-sorted lines generated in-house, F27Ab (from abdominal skin of a female 27-year-old donor) and F48Ab (from abdominal skin of a female 48-year-old donor). The data are from a single experiment and the error bars therefore show technical rather than biological replicates, in contrast to Figure 1. In Supplementary Figure 2, the line with the highest α-SMA expression in the absence of TGF-β1, c24, showed a significant increase in response to TGF-β1. Supplementary Figure 2 also shows that c64a α-SMA expression was low compared to the two in-house lines, as well as to the commercial lines in Figure 1B.

In conclusion, we have identified a commercial line of human skin fibroblasts that did not express α-SMA even following TGF-β1 treatment. This line therefore represents a unique tool with which to explore fibroblast heterogeneity.

### Fibroblast Gene Expression Profiling

To explore the characteristics of c64a cells in more depth, the gene expression profile of the c64a line was compared with three non-sorted fibroblast lines that were isolated in-house and assayed at passage 7-10: F22Br (from breast skin of a female 22-year-old donor), M50F (from facial skin of a male 50-year-old donor), and F60Br (from breast skin of a female 60-year-old donor). Cells were cultured in DMEM with 10% FBS for 4 days, then transferred to low (1%) serum DMEM for 24 h, and treated with or without TGF-β1 for a further 12 or 24 h prior to RNA extraction.

**FIGURE 2:**
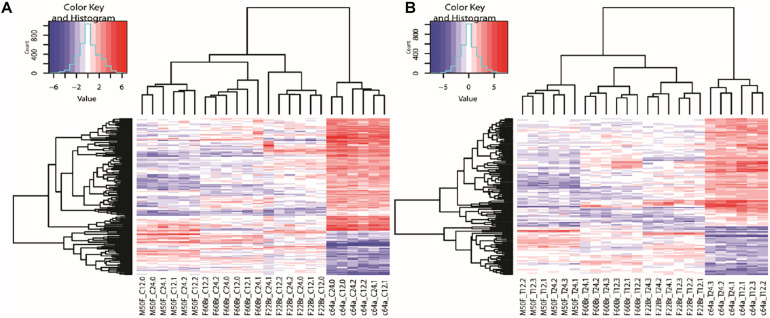
Clustering of genes differentially expressed in c64a fibroblasts. **(A)** 150 probes representing 140 differentially expressed genes at 12 or 24 h in low serum DMEM. **(B)** 126 probes representing 115 differentially expressed genes after treatment with TGF-β1 for 12 or 24 h in low serum DMEM. LogFC ±2, adjusted *p* < 0.05. Fibroblast lines were F22Br, M50F, F60Br, and PromoCell c64a. Code: Non-treated (C) or TGF-β1-treated (T) were analysed at 12 or 24 h in three independent experiments (0, 1, 2 in **A**; 1, 2, 3 in **B**).

Microarray data were generated from triplicate experiments. Data from each experiment were normalised within experiments using GeneSpring and analysed using RStudio. Supplementary Table 4 shows the genes that were significantly up or downregulated in all four fibroblast lines on TGF-β1 treatment. A list of significantly differentially expressed genes in c64a cells compared to the other cell lines (Supplementary Table 5) was used to form heatmaps (Figure 2) as well as for Gene Ontology (GO) terms and Ingenuity Pathway Analysis (IPA). Figure 2 shows genes whose expression was significantly (*p* < 0.05) increased (red) or decreased (blue) with a log fold change (logFC) of ±2 in c64a fibroblasts compared to the other lines in the absence (Figure 2A) or presence (Figure 2B) of TGF-β1.

Supplementary Figure 3 shows the top 10 GO terms for non-treated and TGF-β1-treated c64a cells compared with the other cell lines, F22Br, M50F, and F60Br. In the case of non-treated cells, the most significant processes that distinguished c64a cells included those related to development, differentiation, morphogenesis, and proliferation. Similar terms were found in the GO terms for TGF-β1-treated cells, with the additional term “Immune System Process.” c64a cells were selected by flow cytometry on the basis of CD90 expression prior to culture, whereas F22Br, M50F, and F60Br were not. Nevertheless, M50F and c64a cells expressed similar levels of CD90 mRNA whether cultured in the presence or absence of TGF-β1 (Supplementary Figure 4).

When we compared the genes that were increased in expression in all four lines in response to TGF-β1, the genes that were more than fivefold increased were KN motif and ankyrin repeat domains 4 (KANK4) and early growth response 2 (EGR2) (Supplementary Table 4). KANK4 expression is associated with fibroblast contractility (Haydont et al., 2019), which is stimulated by TGF-β1 (Vallée and Lecarpentier, 2019), while EGR2 is a known transcriptional target of TGF-β1 (Fang et al., 2011). These observations suggest that the lack of α-SMA+ c64a cells was not due to a defect in TGF-β1 responsiveness.

IPA (Supplementary Figure 5) shows the top network for c64a differentially expressed genes under non-treated conditions and following TGF-β1 treatment. ERK1/2 was at the centre of the c64a specific signalling network in both cases. Under non-treated conditions, there was significant downregulation of WNT signalling, while when c64a cells were treated with TGF-β1, there was notable upregulation of several chemokines, including C-X-C motif chemokine ligand 1 (CXCL1)-3 and CXCL6.

In summary, by comparing the gene expression profiles of the c64a line with three in-house fibroblast lines, we could conclude that the unique properties of c64a cells did not reflect a defect in TGF-β1 responsiveness and were unlikely to be due to selection of c64a cells by CD90 expression prior to culture. These findings led us to examine the differentially expressed genes in more detail.

### Mapping of Differentially Expressed Genes

The most highly differentially expressed genes in non-treated and TGF-β1 treated c64a fibroblasts are shown in Supplementary Table 5. Insulin-like growth factor 1 (IGF1) was the gene with the greatest increase in expression, while Wnt family member 4 (WNT4) was one of the genes most decreased in expression compared to other fibroblast lines, in the presence or absence of TGF-β1. Asporin (ASPN) is an endogenous TGF-β1 inhibitor that was significantly decreased in expression in both datasets. CXCL1 expression increased in TGF-β1-treated c64a, but not in the other lines, which is of interest given the differential expression of chemokines in mouse and human fibroblast subpopulations (Philippeos et al., 2018; Tabib et al., 2018).

**FIGURE 3:**
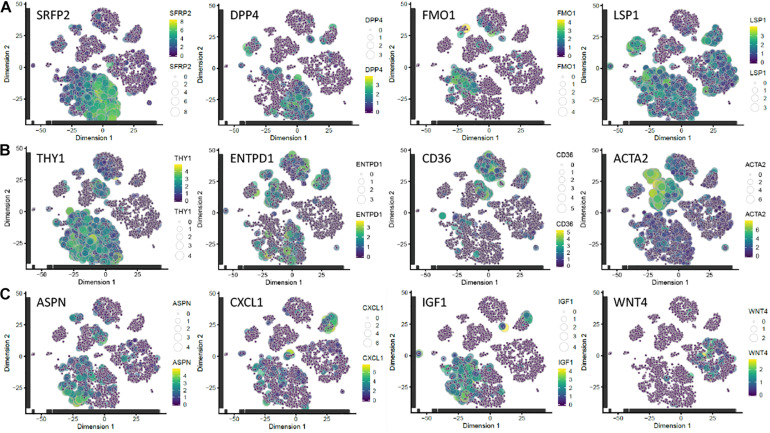
Mapping of candidate markers onto scRNAseq dataset of Tabib et al. (2018). **(A)** Subpopulations of fibroblasts identified by expression of SFRP2/DPP4 and FMO1/LSP1. **(B)** Subpopulations expressing THY1 (CD90; a pan-fibroblast marker); ENTPD1 (CD39), a papillary fibroblast marker; CD36, a reticular fibroblast marker (Philippeos et al., 2018); and ACTA2 (α-SMA). **(C)** Genes differentially expressed in c64a fibroblasts. Transcript expression of the selected markers is overlaid on the 2D t-SNE space of human fibroblasts in the dataset of Tabib et al. (2018). Size and colour represent Log10(TPM) normalised expression values. t-SNE, t-distributed stochastic neighbour embedding; TPM, transcripts per million.

To determine whether ASPN, CXCL1, IGF1, and WNT4 were differentially expressed in human fibroblasts isolated directly from skin, we mapped their expression onto the single cell RNAseq dataset of Tabib et al. (2018). There was no single cluster of freshly-isolated fibroblasts with the characteristics of high IGF1 and CXCL1 and low ASPN and WNT4 expression (Figure 3), and the markers did not map specifically to the SFRP2 or FMO1/LSP1 fibroblast subsets described previously (Tabib et al., 2018). There were very few WNT4+ fibroblasts, and they mapped to a distinct population of CD90- fibroblasts (Figure 3). Other fibroblast markers—DPP4 (CD26), ENTPD1 (CD39), CD36, and ACTA2 (α-SMA)—are shown for comparison. The α-SMA+ fibroblast population lacked CXCL1, IGF1, and WNT4 expression, but included some ASPN+ cells (Figure 3).

We conclude that the most highly differentially expressed genes in c64a cells are ASPN, CXCL1, IGF1, and WNT4. These markers do not define a distinct population of fibroblasts freshly isolated from human skin but provide a tool for functional analysis of the unique properties of c64a cells.

### Functional Effects of IGF1 and WNT4 Knockdown

**FIGURE 4:**
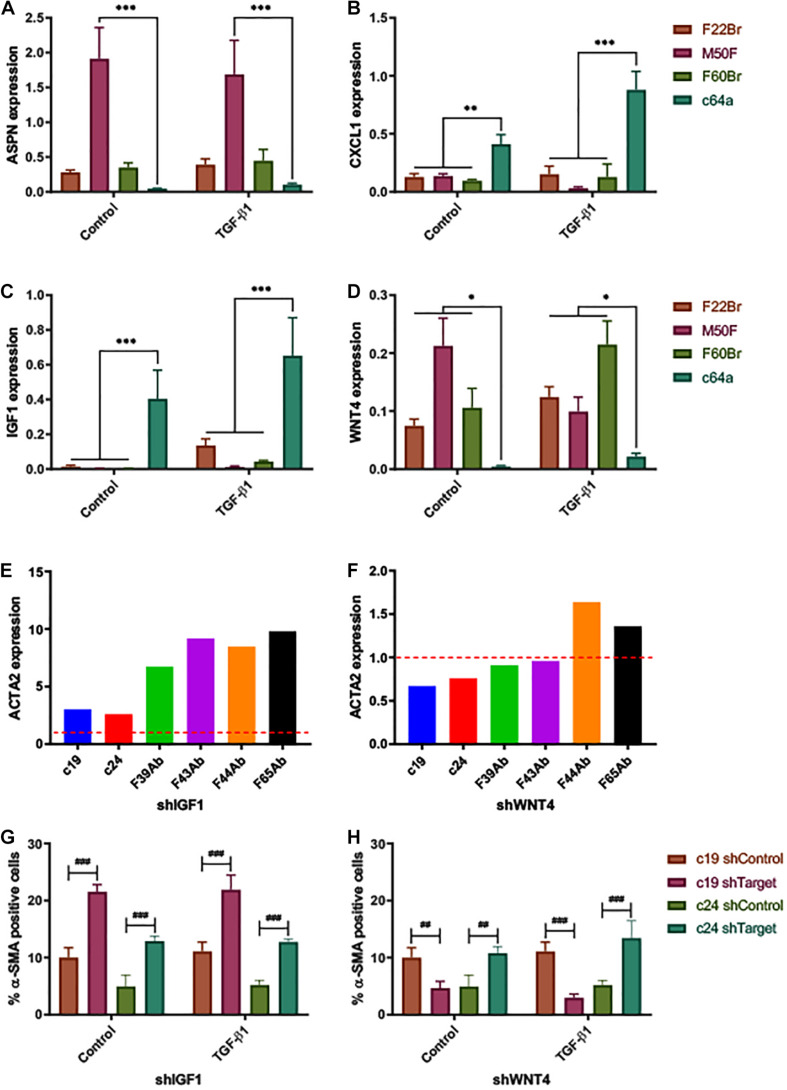
Expression of fibroblast markers. ΔCq expression of **(A)** ASPN, **(B)** CXCL1, **(C)** IGF1, and **(D)** WNT4, 24 h after treatment with TGF-β1 or low serum DMEM (control). Expression relative to reference gene (PPIA). Error bars represent SD of mean values from three independent experiments (*n* = 3). Two-way ANOVA comparing c64a with the fibroblast lines indicated. Dunnett’s multiple comparisons test. *p < 0.05, **p < 0.01, and ***p < 0.001. TGF-β1 treatment **(D)** had a significant effect on WNT4 expression in the case of M50F and F60Br (*p* < 0.001, Sidak’s multiple comparisons test) but not F22Br or c64a. α-SMA (ACTA2) gene expression levels in NHDF expressing shIGF1 **(E)** and shWNT4 **(F)**. Bars represent ΔΔCq expression values for each NHDF line, relative to non-coding control (shControl; —), normalised to the reference gene (18S). Effect of IGF1 knockdown (shIGF1, **G**) and WNT4 knockdown (shWNT4, **H**) on PromoCell c19 and c24 lines. Control cells (low serum DMEM) and TGF-β1-treated cells (10 ng/ml, in low serum DMEM) were assayed for % α-SMA positive cells. Error bars represent SD of mean values of triplicate wells in 3 × 96-well microplates (*n* = 1 experiment). Two-way ANOVA comparing targeted NHDF (shTarget) with own non-coding control. Tukey’s multiple comparisons test. ^##^*p* < 0.01, and ^###^*p* < 0.001.

To validate the gene expression profiling data, we performed Fluidigm TaqMan qPCR on the non-treated and TGF-β1-treated fibroblast lines that were used for the microarray analysis (Figures 4A–D). Consistent with the microarray data, ASPN and WNT4 were decreased and CXCL1 and IGF1 were increased in c64a fibroblasts compared to F22Br, M50F, and F60Br fibroblasts.

To determine the functional significance of the genes identified by microarray analysis, we introduced shRNAs into fibroblasts via lentiviral infection (Supplementary Figure 6). The degree of knockdown varied. With shIGF1 and shWNT4, a reduction in expression was observed in all infected primary cells (6/6 lines tested). Cells in which there was knockdown of CXCL1 (4/6 lines tested) could not be expanded in culture, and ASPN was not pursued further because the effects of knockdown were inconsistent (i.e., achieved in 4/6 lines tested). IGF1 knockdown (shIGF1) led to an increase in α-SMA expression, both at mRNA (qPCR, Figure 4E) and protein levels (immunofluorescence labelling, Figure 4G) in the commercial c19 and c24 fibroblast lines, even though the degree of knockdown was low in c19 cells (Supplementary Figure 6C). shWNT4 knockdown decreased the proportion of α-SMA+ cells in the c19 line, both in the presence and absence of TGF-β1; in contrast, it had the opposite effect in c24 cells (Figure 4H). These findings implicate a role for high IGF1 expression in preventing myofibroblast formation, although this was not tested in c64a cells.

**FIGURE 5:**
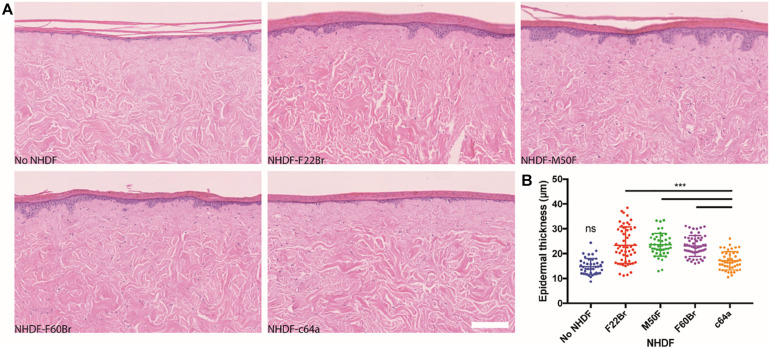
Epidermal thickness in skin reconstitution models. H&E stained sections of de-epidermised dermis (DED), seeded with keratinocytes and grown at the air-liquid interface for 2 weeks **(A)**. The dermis was reconstituted with the NHDF indicated or did not contain fibroblasts. Scale bar: 200 μm. **(B)** Quantitation of epidermal thickness. One-way ANOVA comparing c64a-reconstituted skin with other conditions. Tukey’s multiple comparisons test. ***p < 0.001. Data are from three biological replicates per condition. Two histological slides of each DED, containing four sections per slide, were analysed (eight data points per condition), corresponding to a total of 30 slides and 120 H&E stained sections. Sections were imaged using the NanoZoomer 2.0RS at 20x magnification. Error bars represent SD.

To assay the effects in a skin reconstitution assay, control and knockdown fibroblasts were introduced into de-epidermised human dermis and cultured at the air-liquid interface with human keratinocytes. Epidermal stratification and differentiation could readily be assessed by H&E staining (Figure 5). Immunolabelling with an antibody to keratin 14 stained all the viable epidermal layers See comments in supplementary, while immunolabelling with an antibody to vimentin labelled fibroblasts. The papillary (upper) and reticular dermis can be distinguished in H&E stained sections because of the relative absence of mature collagen fibres in the papillary dermis (Philippeos et al., 2018). The distribution of fibroblasts was not confined to the upper dermis See comments in supplementary. Introduction of F22Br, M50F, or F60Br fibroblasts led to an increase in epidermal thickness compared to cultures that lacked any fibroblasts, as already reported (Wang et al., 2012; Philippeos et al., 2018). In contrast, there was no significant difference between the thickness of epidermis cultured on de-cellularised dermis lacking fibroblasts and dermis containing c64a fibroblasts (Figure 5).

**FIGURE 6:**
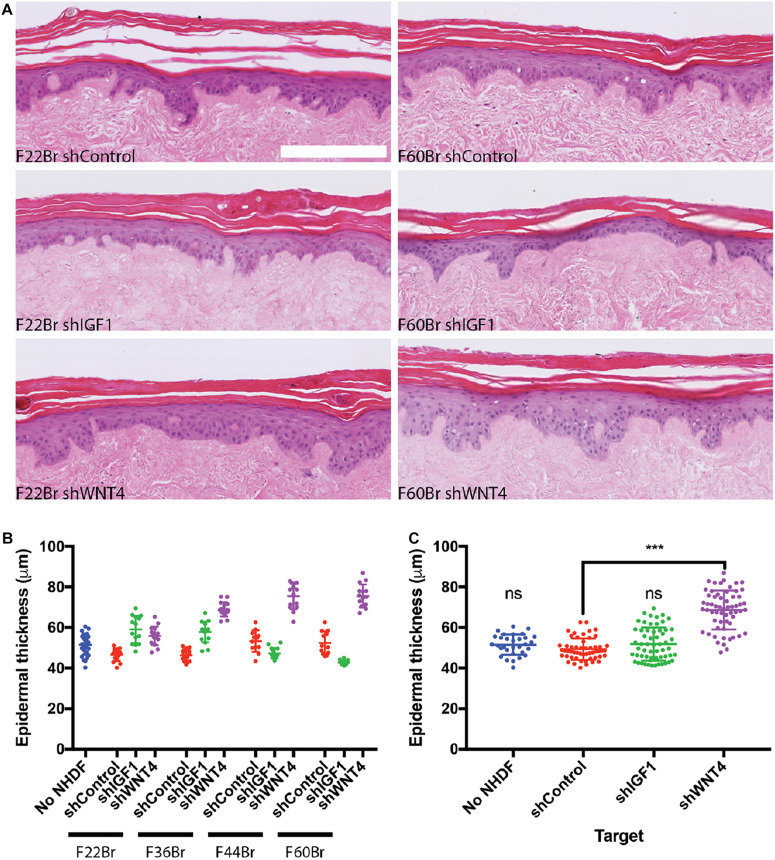
Effect of shRNA-based lentiviral knockdown of IGF1 and WNT4 in skin reconstitution models. **(A)** Examples of H&E stained sections. Scale bar: 250 μm. **(B,C)** Quantitation of epidermal thickness. **(B)** Individual lentiviral-targeted NHDF: female 22-year-old breast skin (F22Br), female 36-year-old breast skin (F36Br), female 44-year-old breast skin (F44Br), and 60-year-old breast skin (F60Br). **(C)** Pooled data from the four individual lines in **(B)**. One-way ANOVA, Dunnett’s multiple comparisons test compared with shControl. Error bars represent SD of data points. ***p < 0.001. Data collection as in Figure 5, except that *n* = 1 DED for each lentiviral-targeted cell line and *n* = 2 for control (no NHDF) DEDs.

We next examined the effect of knocking down IGF1 and WNT4 in four in-house fibroblast lines: F22Br, F36Br, F44Br, and F60Br (Figure 6). Knockdown fibroblasts were cultured on tissue culture plastic with puromycin selection for 3 days prior to injection into DED and were not exposed to puromycin during skin reconstitution. When the data were combined from all four lines, there was no significant effect of IGF1 knockdown on epidermal thickness, although in two of the lines (F22Br and F36Br), there was a trend towards increased thickness (Figure 6B). In contrast, knockdown of WNT4 led to an increase in epidermal thickness in all four lines, whether analysed individually (Figure 6B) or combined (Figure 6C).

**FIGURE 7:**
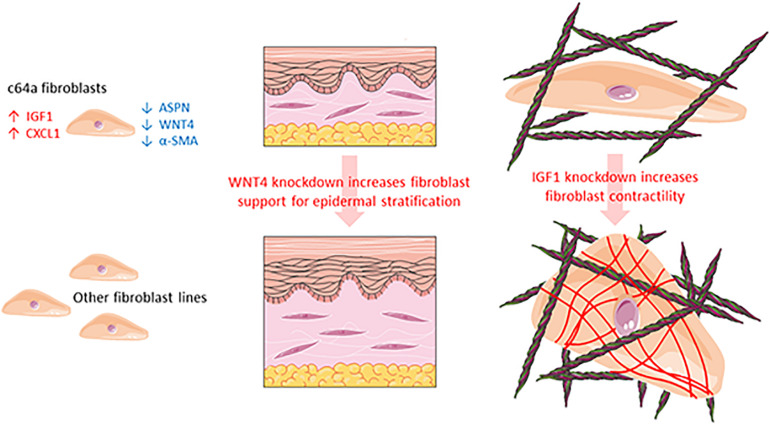
Schematic summarising the different properties of c64a fibroblasts compared to other fibroblast lines.

In summary, we were able to confirm differential expression of ASPN, CXCL1, IGF1, and WNT4 in c64a cells and obtain evidence that IGF1 and WNT4 contribute to two distinct dermal functions: myofibroblast formation and epidermal maintenance, respectively (Figure 7).

## Discussion

In the past 5 years, we have isolated fibroblasts from surgical waste skin of multiple adult donors and observed differences in cell growth and morphology that we could not account for simply based on the age, sex, or body site of the donors (Philippeos et al., unpublished). Studies in mice demonstrate that even within a single body site, dermal fibroblasts have different embryological origins and functions (Driskell et al., 2013; Rinkevich et al., 2014, 2015). In addition, single-cell RNAseq of fibroblasts isolated directly from human dermis reveals the existence of multiple fibroblast populations with different functions (Philippeos et al., 2018; Tabib et al., 2018; Solé-Boldo et al., 2020; Reynolds et al., 2021). These observations prompted us to explore whether or not we could use differences between NHDF lines that had been established in culture for six or more passages as a novel approach to identify candidate regulators of fibroblast function (Figure 7).

By comparing four different fibroblast lines obtained from a commercial supplier, we identified one (c64a) that failed to express α-SMA in the presence or absence of TGF-β1. We identified four genes (ASPN, CXCL1, IGF1, and WNT4) as being most highly differentially expressed in the c64a line: c64a cells expressed higher levels of CXCL1 and IGF1 and lower levels of ASPN and WNT4 than the other three lines (Figure 7). We showed that knockdown of IGF1 in six independent adult human fibroblast lines (two commercial, four in-house) increased α-SMA expression. Although the degree of knockdown achieved varied between lines, this did not correlate with the increase in α-SMA expression. We also found that knockdown of WNT4 in four in-house fibroblast lines led to improved epidermal reconstitution.

Although changes in fibroblast gene expression and function with ageing have been described extensively (Schulze et al., 2010; Lago and Puzzi, 2019), we could not account for the unusual features of c64a cells on that basis because in our assays c64a cells were distinguishable from age-matched fibroblasts from other donors. In contrast to our in-house lines, the commercial lines were flow sorted on the basis of CD90 expression prior to plating and expansion in culture. Although Korosec et al. (2019) have reported that CD90 is a marker of reticular fibroblasts, c64a cells differed from both CD90-sorted (commercial) and unsorted (in-house) lines.

All four differentially expressed genes identified in our screen are implicated in skin homeostasis. CXCL1 promotes neutrophil infiltration during wound healing (reviewed by Gillitzer and Goebeler, 2001). CXCL1 expression is increased in keloids and promotes keratinocyte migration (Gillitzer and Goebeler, 2001). ASPN expression is increased in fibroblasts of tumour stroma, and loss of ASPN prevents self-renewal of mesenchymal stromal cells (Hughes et al., 2019).

Insulin-like growth factor 1 production is reduced in aged skin fibroblasts, which has a negative impact on the epidermal response to UV-mediated DNA damage (Kemp et al., 2017). All-trans retinoic acid increases IGF1 expression and IGF receptor signalling, one of the mechanisms by which all-trans retinoic acid improves the properties of photo-aged human skin by upregulating prolidase-dependent collagen synthesis (Shim et al., 2012).

Wnt4 is induced by hypoxia and is increased in expression during wound healing in mouse skin (Carre et al., 2018). Wnt4 expression is increased upon TGF-β1 treatment of mouse fibroblasts (Colwell et al., 2006; Liu et al., 2020). Wnt4 expression is increased in post-natal wound healing and hypertrophic scars, inhibiting TGF-β1-induced myofibroblast differentiation and α-SMA expression (Colwell et al., 2006; Liu et al., 2020). However, TGF-β1 treatment had no consistent effect on WNT4 expression in the human cell lines we tested, either increasing, decreasing, or having no effect on levels, depending on the line (Figure 4). ERK1/2 was at the centre of the signalling pathways that were upregulated in c64a cells, consistent with the finding that Wnt4 treatment can block ERK phosphorylation (Liu et al., 2020).

The genes that distinguish c64a cells from other skin fibroblasts do not map neatly onto a distinct subpopulation of cells *in vivo*, leading us to propose that their properties have evolved in culture, potentially by a combination of selection and the effect of culture on gene expression (Philippeos et al., 2018). In re-analysing the dataset of Tabib et al. (2018), cells with high levels of ASPN, IGF1 and CXCL1 and low levels of WNT4 map to the major CD90+ cluster and are relatively low in SRFP2 and high in FMO1/LSP1. SFRP2+ dermal fibroblasts are reported to be small, elongated cells, in contrast to FMO1+/LSP1+ cells, which are associated with inflammation and matrix deposition. The different subsets do not have a distinct spatial distribution (Tabib et al., 2018), in contrast to other subpopulations which map specifically to papillary (CD39) or reticular (CD36) dermis (Philippeos et al., 2018). Nevertheless, the cluster with highest α-SMA (ACTA2) is low in IGF1, as would be predicted from our functional studies. It is striking that ACTA2 does not emerge as a marker of any of the major fibroblast subsets recently identified by single cell RNA sequencing (Solé-Boldo et al., 2020; Reynolds et al., 2021), reinforcing the observation that the properties of fibroblasts change significantly in culture (Philippeos et al., 2018).

A detailed characterisation of human dermal fibroblast subpopulations offers considerable potential to develop novel strategies to treat conditions such as fibrotic scarring or epidermal thinning (Lynch and Watt, 2018). A transient decrease in IGF1 expression might improve cell and ECM contractility without the long-term consequences of excess myofibroblast accumulation (Tomasek et al., 2002). Inhibiting WNT4 could have a beneficial effect on epidermal maintenance. Nevertheless, it is intriguing that even though c64a fibroblasts expressed low levels of WNT4 they were very ineffective at promoting epidermal stratification (Figure 5). This suggests that additional analysis of the c64a gene expression profile and functional validation of additional candidate genes such as ASPN and CXCL1 would be worthwhile.

## Conclusion

Although the c64a cell line does not appear to correspond to a known fibroblast subpopulation *in vivo*, it has, nevertheless, demonstrated the utility of exploring differences between fibroblasts cultured from different donors. Further studies involving IGF1 and WNT4 overexpression in cultured human fibroblasts would clearly be of interest. It would be valuable to see whether the reduced ability of c64a cells to support epidermal stratification can be rescued by simultaneously knocking down IGF1 and overexpressing WNT4 as this would reveal whether there is a direct connection between IGF1 and WNT4 in epidermal maintenance. Furthermore, *in vivo* wounding experiments with dermal specific IGF1-KO or WNT4-KO mice would be useful to more conclusively address the functional roles of IGF1 and WNT4 as they would enable measurement of α-SMA+ myofibroblast transition with granulation tissue formation, speed of epidermal closure, and any effect on the extent of fibrosis in the repaired tissue.

## Data Availability Statement

The datasets presented in this study can be found in online repositories. The names of the repository/repositories and accession number(s) can be found in the article/Supplementary Material.

## Ethics Statement

The human skin fibroblasts used in this study were either obtained from a commercial supplier (PromoCell; C-12302) or from surgical waste skin obtained with appropriate ethical approval (REC 14/NS/1073) from the Department of Plastic and Reconstructive Surgery, St George’s Hospital, London. The patients/participants provided their written informed consent to participate in this study.

## Author Contributions

OC, RS, and FW: conceptualisation. OC, MT, JS, and DH: data curation. OC, CP, and BO: formal analysis. GJ, RB, RS, and FW: funding acquisition. OC, BL, CP, and BO: investigation. OC, BL, CP, BO, MT, DH, and JS: methodology. GJ, RB, RS, and FW: project administration. GJ, RB, RS, BO, CP, and FW: supervision. OC, BL, MT, BO, and CP: visualisation. OC and FW: writing—original draft. BO, OC, and FW: writing—review and editing. All authors contributed to the article and approved the submitted version.

## Conflict of Interest

DH, GJ, and RB are employees of Unilever. FW is on secondment as Executive Chair, Medical Research Council. The remaining authors declare that the research was conducted in the absence of any commercial or financial relationships that could be construed as a potential conflict of interest.

## Footnotes

1 https://www.r-project.org/

2 https://www.ncbi.nlm.nih.gov/geo/

## References

[B1] Ashburner M. Ball C. A. Blake J. A. Botstein D. Butlerm H. Cherry J. M. (2000). Gene ontology: tool for the unification of biology. *Nat. Genet.* 25 25–29. 10802651 10.1038/75556 PMC3037419

[B2] Bajouri A. Orouji Z. Taghiabadi E. Nazari A. Shahbazi A. Fallah N. (2020). Long-term follow-up of autologous fibroblast transplantation for facial contour deformities, a non-randomized phase IIa clinical trial. *Cell J.* 22 75–84. 31606970 10.22074/cellj.2020.6340 PMC6791067

[B3] Ben-David U. Beroukhim R. Golub T. R. (2019). Genomic evolution of cancer models: perils and opportunities. *Nat. Rev. Cancer* 19 97–109. 10.1038/s41568-018-0095-3 30578414 PMC6493335

[B4] Carre A. L. Hu M. S. James A. W. Kawai K. Galvez M. G. Longaker M. T. (2018). β-catenin-dependent Wnt signaling: a pathway in acute cutaneous wounding. *Plast. Reconstr. Surg.* 141 669–678. 10.1097/prs.0000000000004170 29481398 PMC6545117

[B5] Clark R. A. McCoy G. A. Folkvord J. M. McPherson J. M. (1997). TGF-beta 1 stimulates cultured human fibroblasts to proliferate and produce tissue-like fibroplasia: a fibronectin matrix-dependent event. *J. Cell. Physiol.* 170 69–80. 10.1002/(sici)1097-4652(199701)170:1<69::aid-jcp8>3.0.co;2-j 9012786

[B6] Colwell A. S. Krummel T. M. Longaker M. T. Lorenz H. P. (2006). Wnt-4 expression is increased in fibroblasts after TGF-beta1 stimulation and during fetal and postnatal wound repair. *Plast. Reconstr. Surg.* 117 2297–2301. 10.1097/01.prs.0000218708.16909.31 16772932

[B7] Driskell R. R. Clavel C. Rendl M. Watt F. M. (2011). Hair follicle dermal papilla cells at a glance. *J. Cell Sci.* 124 1179–1182. 10.1242/jcs.082446 21444748 PMC3115771

[B8] Driskell R. R. Juneja V. R. Connelly J. T. Kretzschmar K. Tan D. W. Watt F. M. (2012). Clonal growth of dermal papilla cells in hydrogels reveals intrinsic differences between Sox2-positive and -negative cells in vitro and in vivo. *J. Invest. Dermatol.* 132 1084–1093. 10.1038/jid.2011.428 22189784 PMC3306894

[B9] Driskell R. R. Lichtenberger B. M. Hoste E. Kretzschmar K. Simons B. D. Charalambous M. (2013). Distinct fibroblast lineages determine dermal architecture in skin development and repair. *Nature* 504 277–281. 10.1038/nature12783 24336287 PMC3868929

[B10] Fang F. Ooka K. Bhattachyya S. Wei J. Wu M. Du P. (2011). The early growth response gene Egr2 (alias Krox20) is a novel transcriptional target of transforming growth factor-β that is up-regulated in systemic sclerosis and mediates profibrotic responses. *Am. J. Pathol.* 178 2077–2090. 10.1016/j.ajpath.2011.01.035 21514423 PMC3081194

[B11] Fujiwara H. Ferreira M. Donati G. Marciano D. K. Linton J. M. Sato Y. (2011). The basement membrane of hair follicle stem cells is a muscle cell niche. *Cell* 144 577–589. 10.1016/j.cell.2011.01.014 21335239 PMC3056115

[B12] Gillitzer R. Goebeler M. (2001). Chemokines in cutaneous wound healing. *J. Leukoc. Biol.* 69 513–521. 11310836

[B13] Grinnell F. (1994). Fibroblasts, myofibroblasts, and wound contraction. *J. Cell Biol.* 124 401–404. 10.1083/jcb.124.4.401 8106541 PMC2119916

[B14] Guerrero-Juarez C. F. Dedhia P. H. Jin S. Ruiz-Vega R. Ma D. Liu Y. (2019). Single-cell analysis reveals fibroblast heterogeneity and myeloid-derived adipocyte progenitors in murine skin wounds. *Nat. Commun.* 10:650. 10.1038/s41467-018-08247-x PMC6368572 30737373

[B15] Hagood J. S. Prabhakaran P. Kumbla P. Salazar L. MacEwen M. W. Barker T. H. (2005). Loss of fibroblast Thy-1 expression correlates with lung fibrogenesis. *Am. J. Pathol.* 167 365–379. 10.1016/s0002-9440(10)62982-3 16049324 PMC1603564

[B16] Haydont V. Neiveyans V. Zucchi H. Fortunel N. O. Asselineau D. (2019). Genome-wide profiling of adult human papillary and reticular fibroblasts identifies ACAN, Col XI α1, and PSG1 as general biomarkers of dermis ageing, and KANK4 as an exemplary effector of papillary fibroblast ageing, related to contractility. *Mech. Ageing Dev.* 177 157–181. 10.1016/j.mad.2018.06.003 29913199

[B17] Hinz B. Celetta G. Tomasek J. J. Gabbiani G. Chaponnier C. (2001). Alpha-smooth muscle actin expression upregulates fibroblast contractile activity. *Mol. Biol. Cell* 12 2730–2741. 10.1091/mbc.12.9.2730 11553712 PMC59708

[B18] Hu P. Barker T. H. (2019). Thy-1 in integrin mediated mechanotransduction. *Front. Cell Dev. Biol.* 7:22. 10.3389/fcell.2019.00022 30859101 PMC6397864

[B19] Hughes P. Marshall D. Reid Y. Parkes H. Gelber C. (2007). The costs of using unauthenticated, over-passaged cell lines: how much more data do we need? *Biotechniques* 43 575–586. 10.2144/000112598 18072586

[B20] Hughes R. M. Simons B. W. Khan H. Miller R. Kugler V. Torquato S. (2019). Asporin restricts mesenchymal stromal cell differentiation, alters the tumor microenvironment, and drives metastatic progression. *Cancer Res.* 79 3636–3650. 10.1158/0008-5472.can-18-2931 31123087 PMC6734938

[B21] Ichida J. K. Blanchard J. Lam K. Son E. Y. Chung J. E. Egli D. (2009). A small-molecule inhibitor of TGF-beta signaling replaces Sox2 in reprogramming by inducing nanog. *Cell Stem Cell* 5 491–503. 10.1016/j.stem.2009.09.012 19818703 PMC3335195

[B22] Kemp M. G. Spandau D. F. Travers J. B. (2017). Impact of age and insulin-like growth factor-1 on DNA damage responses in UV-irradiated human skin. *Molecules* 22:356. 10.3390/molecules22030356 28245638 PMC5432641

[B23] Klingberg F. Chow M. L. Koehler A. Boo S. Buscemi L. Quinn T. M. (2014). Prestress in the extracellular matrix sensitizes latent TGF-β1 for activation. *J. Cell Biol.* 207 283–297. 10.1083/jcb.201402006 25332161 PMC4210443

[B24] Korosec A. Frech S. Gesslbauer B. Vierhapper M. Radtke C. Petzelbauer P. (2019). Lineage identity and location within the dermis determine the function of papillary and reticular fibroblasts in human skin. *J. Invest. Dermatol.* 139 342–351. 10.1016/j.jid.2018.07.033 30179601

[B25] Krämer A. Green J. Pollard J. Jr. Tugendreich S. (2014). Causal analysis approaches in Ingenuity Pathway Analysis. *Bioinformatics* 30 523–530. 10.1093/bioinformatics/btt703 24336805 PMC3928520

[B26] Lago J. C. Puzzi M. B. (2019). The effect of aging in primary human dermal fibroblasts. *PLoS One* 14:e0219165. 10.1371/journal.pone.0219165 31269075 PMC6608952

[B27] Liberzon A. Subramanian A. Pinchback R. Thorvaldsdóttir H. Tamayo P. Mesirov J. P. (2011). Molecular signatures database (MSigDB) 3.0. *Bioinformatics* 27 1739–1740. 10.1093/bioinformatics/btr260 21546393 PMC3106198

[B28] Lichtenberger B. M. Mastrogiannaki M. Watt F. M. (2016). Epidermal β-catenin activation remodels the dermis via paracrine signalling to distinct fibroblast lineages. *Nat. Commun.* 7:10537. 10.1038/ncomms10537 PMC4742837 26837596

[B29] Liu J. Wang Y. Pan Q. Su Y. Zhang Z. Han J. (2012). Wnt/β-catenin pathway forms a negative feedback loop during TGF-β1 induced human normal skin fibroblast-to-myofibroblast transition. *J. Dermatol. Sci.* 65 38–49. 10.1016/j.jdermsci.2011.09.012 22041457

[B30] Liu J. Zhao B. Zhu H. Pan Q. Cai M. Bai X. (2020). Wnt4 negatively regulates the TGF-β1-induced human dermal fibroblast-to-myofibroblast transition via targeting Smad3 and ERK. *Cell Tissue Res.* 379 537–548. 10.1007/s00441-019-03110-x 31776823

[B31] Livak K. J. Schmittgen T. D. (2001). Analysis of relative gene expression data using real-time quantitative PCR and the 2(-delta delta C(T)) method. *Methods* 25 402–408. 10.1006/meth.2001.1262 11846609

[B32] Lun A. T. L. McCarthy D. J. Marioni J. C. (2016). A step-by-step workflow for low-level analysis of single-cell RNA-seq data with Bioconductor. *F1000Research* 5:2122. 10.12688/f1000research.9501.2 27909575 PMC5112579

[B33] Lynch M. D. Watt F. M. (2018). Fibroblast heterogeneity: implications for human disease. *J. Clin. Invest.* 128 26–35. 10.1172/jci93555 29293096 PMC5749540

[B34] Mishra A. Oulès B. Pisco A. O. Ly T. Liakath-Ali K. Walko G. (2017). A protein phosphatase network controls the temporal and spatial dynamics of differentiation commitment in human epidermis. *Elife* 6:e27356. 10.7554/eLife.27356 PMC5667932 29043977

[B35] Oida T. Weiner H. L. (2010). Depletion of TGF-β from fetal bovine serum. *J. Immunol. Methods.* 362 195–198. 10.1016/j.jim.2010.09.008 20837018 PMC2989462

[B36] Petrof G. Martinez-Queipo M. Mellerio J. E. Kemp P. McGrath J. A. (2013). Fibroblast cell therapy enhances initial healing in recessive dystrophic epidermolysis bullosa wounds: results of a randomized, vehicle-controlled trial. *Br. J. Dermatol.* 169 1025–1033. 10.1111/bjd.12599 24032424

[B37] Philippeos C. Telerman S. B. Oulès B. Pisco A. O. Shaw T. J. Elgueta R. (2018). Spatial and single-cell transcriptional profiling identifies functionally distinct human dermal fibroblast subpopulations. *J. Invest. Dermatol.* 138 811–825. 10.1016/j.jid.2018.01.016 29391249 PMC5869055

[B38] Rashidghamat E. McGrath J. A. (2017). Novel and emerging therapies in the treatment of recessive dystrophic epidermolysis bullosa. *Intractable Rare Dis. Res.* 6 6–20. 10.5582/irdr.2017.01005 28357176 PMC5359356

[B39] Reynolds G. Vegh P. Fletcher J. Poyner E. F. M. Stephenson E. Goh I. (2021). Developmental cell programs are co-opted in inflammatory skin disease. *Science* 371:eaba6500. 10.1126/science.aba6500 PMC7611557 33479125

[B40] Rinkevich Y. Montoro D. T. Muhonen E. Walmsley G. G. Lo D. Hasegawa M. (2014). Clonal analysis reveals nerve-dependent and independent roles on mammalian hind limb tissue maintenance and regeneration. *Proc. Natl. Acad. Sci. U.S.A.* 111 9846–9851. 10.1073/pnas.1410097111 24958860 PMC4103362

[B41] Rinkevich Y. Walmsley G. G. Hu M. S. Maan Z. N. Newman A. M. Drukker M. (2015). Skin fibrosis. Identification and isolation of a dermal lineage with intrinsic fibrogenic potential. *Science* 348:aaa2151. 10.1126/science.aaa2151 25883361 PMC5088503

[B42] Rognoni E. Watt F. M. (2018). Skin cell heterogeneity in development, wound healing, and cancer. *Trends Cell Biol.* 28 709–722. 10.1016/j.tcb.2018.05.002 29807713 PMC6098245

[B43] Schulze C. Wetzel F. Kueper T. Malsen A. Muhr G. Jaspers S. (2010). Stiffening of human skin fibroblasts with age. *Biophys. J.* 99 2434–2442. 10.1016/j.bpj.2010.08.026 20959083 PMC2956221

[B44] Shim J. H. Shin D. W. Lee T. R. Kang H. H. Jin S. H. Noh M. (2012). The retinoic acid-induced up-regulation of insulin-like growth factor 1 and 2 is associated with prolidase-dependent collagen synthesis in UVA-irradiated human dermal equivalents. *J. Dermatol. Sci.* 66 51–59. 10.1016/j.jdermsci.2011.12.008 22245250

[B45] Shook B. A. Wasko R. R. Rivera-Gonzalez G. C. Salazar-Gatzimas E. López-Giráldez F. Dash B. C. (2018). Myofibroblast proliferation and heterogeneity are supported by macrophages during skin repair. *Science* 362:eaar2971. 10.1126/science.aar2971 30467144 PMC6684198

[B46] Solé-Boldo L. Raddatz G. Schütz S. Mallm J. P. Rippe K. Lonsdorf A. S. (2020). Single-cell transcriptomes of the human skin reveal age-related loss of fibroblast priming. *Commun. Biol.* 3:188. 10.1038/s42003-020-0922-4 PMC7181753 32327715

[B47] Sorrell J. M. Caplan A. I. (2004). Fibroblast heterogeneity: more than skin deep. *J. Cell Sci.* 117 667–675. 10.1242/jcs.01005 14754903

[B48] Subramanian A. Tamayo P. Mootha V. K. Mukherjee S. Ebert B. L. Gillette M. A. (2005). Gene set enrichment analysis: a knowledge-based approach for interpreting genome-wide expression profiles. *Proc. Natl. Acad. Sci. U.S.A.* 102 15545–15550. 10.1073/pnas.0506580102 16199517 PMC1239896

[B49] Tabib T. Morse C. Wang T. Chen W. Lafyatis R. (2018). SFRP2/DPP4 and FMO1/LSP1 define major fibroblast populations in human skin. *J. Invest. Dermatol.* 138 802–810. 10.1016/j.jid.2017.09.045 29080679 PMC7444611

[B50] Tomasek J. J. Gabbiani G. Hinz B. Chaponnier C. Brown R. A. (2002). Myofibroblasts and mechano-regulation of connective tissue remodelling. *Nat. Rev. Mol. Cell Biol.* 3 349–363. 10.1038/nrm809 11988769

[B51] Vallée A. Lecarpentier Y. (2019). TGF-β in fibrosis by acting as a conductor for contractile properties of myofibroblasts. *Cell Biosci.* 9:98. 10.1186/s13578-019-0362-3 PMC6902440 31827764

[B52] Wang Z. Wang Y. Farhangfar F. Zimmer M. Zhang Y. (2012). Enhanced keratinocyte proliferation and migration in co-culture with fibroblasts. *PLoS One* 7:e40951. 10.1371/journal.pone.0040951 22911722 PMC3401236

