# Supplementary material for: Differential Expression of Insulin-Like Growth Factor 1 and Wnt Family Member 4 Correlates With Functional Heterogeneity of Human Dermal Fibroblasts
Source: Front Cell Dev Biol. 2021 Apr 6;9:628039. doi: 10.3389/fcell.2021.628039 (PMC8056032; doi:10.3389/fcell.2021.628039)
Supplement: Supplementary file 1 [file Data_Sheet_1.docx]

**Supplementary Materials and Methods**

**Cell culture**

NHDF were cultured in complete phenol red-free, low glucose Dulbecco’s modified Eagle medium (DMEM, Sigma-Aldrich) supplemented with 10% FBS (Sigma-Aldrich), 450 μg/ml L-glutamine, 100 U/ml penicillin and 100 μg/ml streptomycin (Sigma-Aldrich) at 37°C in 5% CO_2_. Cells were passaged 1:3 every 4 days and analysed at passages 6-10. Details of all the fibroblast lines presented in the main Figures are given in Supplementary Table 1.

Keratinocytes were cultured in complete FAD medium consisting of three parts DMEM and one part Ham’s F12 medium supplemented with 1.8 x 10^-4^ M adenine, 10% FBS (Life Tech), 450 μg/ml L-glutamine, 5 μg/ml insulin (Sigma-Aldrich), 0.5 μg/ml hydrocortisone (Thermo Scientific), 1 x 10^-10^ M cholera toxin (Sigma-Aldrich), 10 ng/ml epidermal growth factor (PeproTech), 100 U/ml penicillin and 100 μg/ml streptomycin.

All cells were screened at least every two months for mycoplasma and were found to be mycoplasma-negative. STR profiling (Supplementary Table 2) confirmed that the following lines are derived from different donors: c19, c24, c64a, c64b, M50F, F22Br and F60Br.

**High content imaging of fibroblasts**

Fixed cells were washed twice in PBS, permeabilised in 0.2% Triton X-100 in PBS for 5 min, washed twice with PBS and blocked for 1.5 h in blocking buffer (10% bovine serum albumin (Sigma-Aldrich), 0.25% fish skin gelatin (Sigma-Aldrich) in PBS). Cells were stained using a Click-iT EdU imaging kit (Invitrogen) then incubated in α-actin 1A4 primary antibody (Santa Cruz Biotechnology, sc-32251, 1:300 in blocking buffer) overnight at 4°C. After washing in PBS, cells were incubated in Alexa Fluor 594 secondary antibody (Invitrogen, A21203, 1:1000 in blocking buffer), with CellMask deep red stain (1:1000; Invitrogen). Cells were imaged at 10x magnification using the Operetta Mark1 high content imaging system (Perkin Elmer). Harmony software (v.4.1, Perkin Elmer) was used to quantitate % α-SMA positive, % EdU positive and % spindle-shaped cells. The image analysis pipeline is shown in Supplementary Figure 1.

The analysis pipeline for cell morphology first identified nuclei (NucBlue), and then identified the cytoplasm (CellMask). The software was used to classify NHDF into spindle-shaped positive and spindle-shaped negative populations based on individual cell readouts. These readouts were: cell area (μm²), cell roundness, and cell ratio width to length. Small, spindle, elongated cells were classified as positive (green) for spindle-shape morphology. Large, round, shortened cells were classified as negative (red) for spindle-shape morphology. The analysis pipeline was as follows:

Input Image

Stack Processing: Individual Planes

Flatfield Correction: Basic

Find Nuclei

Channel: NucBlue

Method: M

Diameter: 20 μm

Splitting Coefficient: 0.3

Common Threshold: 0.05

Output Population: Nuclei

Calculate Morphology Properties

Population: Nuclei

Region: Nucleus

Method: Standard

Area

Output Properties: Nucleus

Select Population

Population: Nuclei

Method: Filter by Property

Nucleus Area [μm²]: >= 50

Nucleus Area [μm²]: <= 850

Boolean Operations: F1 and F2

Output Population: Nuclei Selected

Find Cytoplasm

Channel: Alexa 647

Nuclei: Nuclei Selected

Method: B

Common Threshold: 0.5

Individual Threshold: 0.2

Select Population 2

Population: Nuclei Selected

Method: Common Filters

Remove Border Objects

Region: Cell

Output Population: Non-border Cells

Calculate Morphology Properties 2

Population: Non-border Cells

Region: Cell

Method: Standard

Area

Roundness

Ratio Width to Length

Output Properties: Morphology Cell

Select Population 3

Population: Non-border

Method: Filter by Property

Morphology Cell Area [μm²]: > 400

Morphology Cell Area [μm²]: < 16000

Operations: F1 and F2

Output Population: Cell Selected

Select Population 4

Population: Cell Selected

Method: Linear Classifier

Number of Classes: 2

Morphology Cell Area [µm^2^]

Morphology Cell Roundness

Morphology Cell Ratio Width to Length

Output Population A: Spindle

Output Population B: Round

**Agilent gene expression microarray and** **Fluidigm 96:96 TaqMan qPCR**

cDNA hybridisation was performed using G3 Human GE 8x60K V2 kit (Agilent, G4851B) in a SureHyb chamber. Feature extraction software (v.9.5.1; Agilent) was used to extract the data. Gene expression files from each experiment were analysed using GeneSpring software (v.14.8, Agilent) and imported into R (v.3.5.1) (https://www.r-project.org/). Gene names were assigned to probes based on the microarray chip that was used. Designs containing the different conditions and factors were developed in RStudio (v.1.0.153). The molecular signatures database (v.6.2) was used to investigate GO terms (Ashburner et al., 2000) associated with differentially expressed genes (Collection 5: GO gene sets) (Subramanian et al., 2005; Liberzon et al., 2011). IPA software (v.01-08, Qiagen) was used to determine causal networks (Krämer et al., 2014). The datasets are deposited in the GEO repository under accession number: GSE140962.

The same RNA was analysed by Fluidigm high throughput qPCR (isolated from NHDF treated for 24 h with or without TGF-β1). SuperScript III first-strand synthesis SuperMix kit (Thermo Scientific) was used to convert RNA to cDNA. For the TaqMan qPCR a 96.96 dynamic array integrated fluidic circuit (IFC) was used, ‘primed’ in the BioMark IFC controller HX (Fluidigm) using control line fluid. Following priming, 40 samples in total and eight standards were plated in duplicate, to cover approximately 10 Cq changes. The chip was loaded using the BioMark IFC controller HX and run on the BioMark System. Cq values for each target gene were processed using the Fluidigm real-time PCR analysis software (v.4.1.3). Results were represented as ΔCq expression.

**Skin reconstitution on de-epidermised dermis (DED)**

Reconstituted skin was fixed in 10% formalin (Sigma-Aldrich) overnight at 4°C and embedded in paraffin using an Excelsior AS tissue processor (Thermo Scientific). 8 µm sections were cut using a Microm HM 355S (Thermo Scientific). Sections were stained with haematoxylin and eosin (H&E) and imaged using a NanoZoomer 2.0RS (Hamamatsu Photonics) at 20x magnification. Two slides per sample, containing four sections per slide, were imaged and quantified as described previously (Mishra et al., 2017; Supplementary Figure 8). The Python analysis script is published by Mishra et al., 2017. Briefly, tissue sections were automatically detected based on k-means segmentation, and colours were rebalanced. The epidermis was then isolated by colour separation (extraction of haematoxylin colour channels by a deconvolution algorithm). After performing automatic thresholding, epidermal area was measured by pixel counting. To measure epidermal thickness, the epidermal area was divided by the epidermal length. The length was determined by a 1-pixel wide reduced-topology skeleton that took into account the undulations of the epidermal/dermal junction.

**Supplementary Figure legends**

**Supplementary Figure 1**. High content imaging pipeline. Fibroblasts were treated in triplicate wells with different agonist/inhibitor in low serum DMEM (TGF-β1 and ALK5 inhibitor, RepSox). Experiments were carried out three times (n=3). (**1**) Input image with NucBlue (blue), EdU (green), α-SMA (orange) and CellMask (red). The analysis pipeline detected cell nuclei based on NucBlue (**2**) and cytoplasm based on CellMask (**3**). Having removed partial cells on the image border (**4**), it was possible to train the software to identify proliferating cells (**5A**), differentiating cells (**5B**) and spindle-shaped cells (**5C**). Positively labelled cells are shown in green. Images are of non-treated (low serum DMEM) c64b cells. Scale bar: 200 µm.

**Supplementary Figure 2**. NHDF phenotypes. NHDF lines were untreated, treated with TGF-β1 (10 ng/ml) and/or RepSox (25 μM) in DMEM containing 1% FBS and assayed for cell number (**A, E, I, M**), differentiation (% α-SMA positive, **B, F, J, N**), proliferation (% EdU positive, **C, G, K, O**) and shape (% spindle-shaped, **D, H, L, P**). Error bars represent SD of mean values in triplicate wells of three 96-well microplates (n=1). Two-way ANOVA comparing TGF-β1/RepSox treated versus own control NHDF (*), or c64a and different cell lines under the same condition (^#^) (Tukey’s multiple comparisons test. * ^#^p<0.05, ** ^##^p<0.01, *** ^###^p<0.001). **A-L** show the individual experiments in Figure 1. **M-P:** PromoCell NHDF-c24 (female 24-year-old breast skin); female 27-year-old abdomen skin (F27Ab); female 48-year-old abdomen skin (F48Ab) and PromoCell NHDF-c64a (female 64-year-old breast skin, donor A).

**Supplementary Figure** **3**. Top 10 GO terms for c64a differentially expressed genes. (**A**) Non-treated (low serum DMEM). (**B**) TGF-β1 treated (in low serum DMEM).

**Supplementary Figure 4**. ΔCq expression of CD90, 24 h after treatment with TGF-β1 or low serum DMEM (control). Expression relative to reference gene (PPIA). Error bars represent SD of mean values from three independent experiments (n=3). Two-way ANOVA comparing c64a with the fibroblast lines indicated. Dunnett’s multiple comparisons test. **p<0.01, ***p<0.001.

**Supplementary Figure 5**. Top interaction networks for differentially expressed c64a genes. (**A**) Non-treated (low serum DMEM). (**B**) TGF-β1 treated (in low serum DMEM). Increased gene expression (red) and decreased gene expression (green) is depicted for each node. Predicted activation (orange), predicted inhibition (blue), inconsistent interactions (yellow), and non-predicted interactions (grey) are also shown. Figure generated using IPA, QIAGEN.

**Supplementary Figure** **6**. Knockdown of ASPN (**A**), CXCL1 (**B**), IGF1 (**C**) and WNT4 (**D**) expression in lentiviral-treated NHDF relative to non-coding control (shControl). % knockdown (1-ΔΔCq expression values *100) for each targeted NHDF.

**Supplementary Figure 7**. Immunofluorescence labelling of reconstituted skin. Sections were labelled for DAPI (nuclear marker; white), keratin 14 (keratinocytes; green) and vimentin (fibroblasts; red). Control (no NHDF); female 22-year-old breast skin (F22Br); male 50-year-old face skin (M50F); female 60-year-old breast skin (F60Br) and PromoCell NHDF-c64a. Scale bar: 200 µm.

**Supplementary Figure 8**. Epidermal thickness measurements in skin reconstitution models. (**A**) Slide overview of four mounted H&E sections (2.5x). (**B**) Apical surface of M50F-injected de-epidermised dermis, seeded with keratinocytes and grown at the air-liquid interface for 2 weeks (20x). (**C**) Epidermal thickness (25 µm) and length (7691 µm) were calculated using a Python script (Mishra et al., 2017) that identified epidermis based on colour gradient. (**D**) Epidermal colour separation (left) of the M50F-injected skin reconstitution model (right). Scale bar: 500 µm.

**Supplementary Tables**

**Supplementary Table 1**. Details of the normal human dermal fibroblasts used to generate data in Figures 1-6. PromoCell NHDF aged 19 (c19, Lot: 4032503.1), 24 (c24, Lot: 4081903.2), 64 donor A (c64a, Lot: 4012203.1) and 64 donor B (c64b, Lot: 3102301.3) years old. All experiments used enzyme-derived NHDF. NHDF ordered on appearance firstly and age secondly, starting with NHDF-c19 and ending with NHDF-F44Br (left-right).

**Supplementary Table 2**. STR profiles of the fibroblast lines indicated.

**Supplementary Table 3**. List of TaqMan qPCR gene expression assays. PPIA, and RPS18 were used as reference genes.

**Supplementary Table 4**. Differentially expressed genes common to NHDF-F22Br, M50F, F60Br and c64a on TGF-β1 treatment (12 and 24 h timepoints combined). LogFC ±2, adjusted p value<0.05. Sorted on logFC.

**Supplementary Table 5**. Genes differentially expressed in NHDF-c64a, sorted on logFC. Adjusted (Adj.) p value<0.05. Asporin (ASPN); C-X-C motif chemokine ligand 1 (CXCL1); Insulin-like growth factor 1 (IGF1); Wnt family member 4 (WNT4). Note: multiple hits for each gene.

**Supplementary Table 1.**

| **NHDF** | **c19** | **c24** | **c64a** | **c64b** | **F22Br** | **M50F** | **F60Br** | **F39Ab** | **F43Ab** | **F44Ab** | **F65Ab** | **F36Br** | **F44Br** |
| --- | --- | --- | --- | --- | --- | --- | --- | --- | --- | --- | --- | --- | --- |
| Gender (F/M) | F | F | F | F | F | M | F | F | F | F | F | F | F |
| Age (years old) | 19 | 24 | 64 | 64 | 22 | 50 | 60 | 39 | 43 | 44 | 65 | 36 | 44 |
| Site of origin | Breast | Breast | Breast | Breast | Breast | Face | Breast | Abdomen | Abdomen | Abdomen | Abdomen | Breast | Breast |
| PromoCell (Lot#) | 4032503.1 | 4081903.2 | 4012203.1 | 3102301.3 |  |  |  |  |  |  |  |  |  |
| NHDF phenotype (Figure 1) | Y | Y | Y | Y |  |  |  |  |  |  |  |  |  |
| NHDF genotype (Figure 2) |  |  | Y |  | Y | Y | Y |  |  |  |  |  |  |
| Fluidigm qPCR (Figure 4, A-D) |  |  | Y |  | Y | Y | Y |  |  |  |  |  |  |
| shRNA qPCR (Figure 4, E-F) | Y | Y |  |  |  |  |  | Y | Y | Y | Y |  |  |
| shRNA phenotype (Figure 4, G-H) | Y | Y |  |  |  |  |  |  |  |  |  |  |  |
| Skin models (Figure 5) |  |  | Y |  | Y | Y | Y |  |  |  |  |  |  |
| shRNA skin models (Figure 6) |  |  |  |  | Y |  | Y |  |  |  |  | Y | Y |

**Supplementary Table 3.**

| **Gene** | **Gene ID** | **Assay #** |
| --- | --- | --- |
| Actin α2/α-SMA | ACTA2 | Hs00426835_g1 |
| Asporin | ASPN | Hs01550901_m1 |
| Cluster of differentiation 90 (THY1) | CD90 | Hs00264235_m1 |
| C-X-C motif chemokine ligand 1 | CXCL1 | Hs00236937_m1 |
| Insulin-like growth factor 1 | IGF1 | Hs01547656_m1 |
| Peptidylprolyl isomerase A | PPIA | Hs04194521_s1 |
| Ribosomal protein S18 | RPS18 | Hs01375212_g1 |
| Wnt family member 4 | WNT4 | Hs01573505_m1 |

**Supplementary Table 4.**

| **LogFC** | **Adj. p value** | **Gene name (ID)** |
| --- | --- | --- |
| 6.84 | 1.71E-21 | KN motif and ankyrin repeat domains 4(KANK4) |
| 6.69 | 1.57E-20 | KN motif and ankyrin repeat domains 4(KANK4) |
| 5.69 | 5.66E-26 | early growth response 2(EGR2) |
| 4.93 | 7.53E-15 | low density lipoprotein receptor class A domain containing 4(LDLRAD4) |
| 4.83 | 2.66E-19 | claudin 14(CLDN14) |
| 4.77 | 4.83E-15 | tetraspanin 2(TSPAN2) |
| 4.55 | 1.58E-20 | adhesion molecule with Ig like domain 2(AMIGO2) |
| 4.49 | 1.64E-22 | interleukin 11(IL11) |
| 4.37 | 3.24E-22 | lnc-RP11-625H11.1.1-3 |
| 4.23 | 3.24E-32 | basic helix-loop-helix family member e40(BHLHE40) |
| 4.18 | 8.48E-15 | frizzled class receptor 8(FZD8) |
| 4.08 | 2.51E-17 | POU class 3 homeobox 2(POU3F2) |
| 4.06 | 1.50E-12 | BTB domain containing 11(BTBD11) |
| 3.95 | 8.65E-22 | NADPH oxidase 4(NOX4) |
| 3.63 | 1.99E-14 | inhibitor of DNA binding 1, HLH protein(ID1) |
| 3.62 | 1.84E-27 | NADPH oxidase 4(NOX4) |
| 3.62 | 4.35E-13 | sphingosine-1-phosphate receptor 5(S1PR5) |
| 3.62 | 6.22E-18 | heparin binding EGF like growth factor(HBEGF) |
| 3.56 | 1.17E-16 | SRY-box 9(SOX9) |
| 3.56 | 1.12E-13 | leukemia inhibitory factor(LIF) |
| 3.55 | 4.27E-28 | xylosyltransferase 1(XYLT1) |
| 3.51 | 5.73E-23 | prostate transmembrane protein, androgen induced 1(PMEPA1) |
| 3.50 | 5.85E-16 | inhibitor of DNA binding 3, HLH protein(ID3) |
| 3.44 | 3.69E-24 | POU class 3 homeobox 2(POU3F2) |
| 3.40 | 1.17E-07 | sclerostin(SOST) |
| 3.38 | 2.62E-14 | tetraspanin 13(TSPAN13) |
| 3.32 | 4.88E-15 | synapse differentiation inducing 1(SYNDIG1) |
| 3.32 | 7.98E-22 | NADPH oxidase 4(NOX4) |
| 3.28 | 1.29E-19 | growth differentiation factor 6(GDF6) |
| 3.28 | 1.11E-10 | long intergenic non-protein coding RNA 1013(LINC01013) |
| 3.20 | 7.17E-20 | dishevelled binding antagonist of beta catenin 1(DACT1) |
| 3.20 | 2.93E-15 | interleukin 21 receptor(IL21R) |
| 3.16 | 4.15E-10 | long intergenic non-protein coding RNA 1013(LINC01013) |
| 3.15 | 3.48E-18 | syndecan 1(SDC1) |
| 3.14 | 3.10E-13 | uncharacterized LOC79160(LOC79160) |
| 3.12 | 5.43E-10 | long intergenic non-protein coding RNA 1013(LINC01013) |
| 3.08 | 1.49E-15 | stathmin domain containing 1(STMND1) |
| 3.04 | 3.91E-11 | spermatogenesis associated 17(SPATA17) |
| 3.03 | 7.26E-14 | PBX/knotted 1 homeobox 2(PKNOX2) |
| 3.03 | 6.05E-09 | transglutaminase 2(TGM2) |
| 2.93 | 1.55E-16 | WNT1 inducible signalling pathway protein 1(WISP1) |
| 2.91 | 2.52E-04 | forkhead box S1(FOXS1) |
| 2.91 | 1.16E-08 | lnc-PRICKLE2-3 |
| 2.91 | 4.04E-06 | hyaluronan synthase 1(HAS1) |
| 2.89 | 2.89E-05 | MyoD family inhibitor(MDFI) |
| 2.87 | 9.01E-10 | archaelysin family metallopeptidase 1(AMZ1) |
| 2.83 | 4.53E-07 | exostosin like glycosyltransferase 1(EXTL1) |
| 2.80 | 7.55E-17 | MIR503 host gene(MIR503HG) |
| 2.80 | 7.96E-16 | MIR503 host gene(MIR503HG) |
| 2.79 | 7.40E-10 | phosphatidic acid phosphatase type 2 domain containing 1A(PPAPDC1A) |
| 2.78 | 1.17E-16 | colony stimulating factor 1 receptor(CSF1R) |
| 2.76 | 1.43E-10 | long intergenic non-protein coding RNA 1013(LINC01013) |
| 2.75 | 7.37E-09 | collagen type X alpha 1 chain(COL10A1) |
| 2.74 | 2.22E-12 | RAS like family 11 member B(RASL11B) |
| 2.74 | 4.09E-07 | TNF alpha induced protein 6(TNFAIP6) |
| 2.68 | 2.41E-11 | LY6/PLAUR domain containing 1(LYPD1) |
| 2.67 | 3.21E-19 | annexin A8-like 1(ANXA8L1) |
| 2.64 | 9.53E-10 | chromosome 15 open reading frame 48(C15orf48) |
| 2.60 | 5.97E-07 | cadherin EGF LAG seven-pass G-type receptor 1(CELSR1) |
| 2.59 | 1.20E-16 | PC-esterase domain containing 1B(PCED1B) |
| 2.55 | 3.02E-10 | F2R like trypsin receptor 1(F2RL1) |
| 2.54 | 1.42E-08 | stimulated by retinoic acid 6(STRA6) |
| 2.52 | 6.70E-16 | immediate early response 3(IER3) |
| 2.51 | 4.64E-10 | opioid binding protein/cell adhesion molecule like(OPCML) |
| 2.51 | 9.90E-22 | cysteine and glycine rich protein 2(CSRP2) |
| 2.50 | 6.93E-13 | EFR3 homolog B(EFR3B) |
| 2.49 | 3.12E-08 | dual specificity phosphatase 26 (putative)(DUSP26) |
| 2.48 | 4.90E-09 | leucine rich repeat neuronal 3(LRRN3) |
| 2.48 | 3.33E-13 | JunB proto-oncogene, AP-1 transcription factor subunit(JUNB) |
| 2.43 | 1.25E-13 | scleraxis bHLH transcription factor(SCX) |
| 2.43 | 2.97E-15 | immediate early response 3(IER3) |
| 2.42 | 9.45E-10 | long intergenic non-protein coding RNA 1013(LINC01013) |
| 2.42 | 2.61E-14 | uncharacterized LOC102724849(LOC102724849) |
| 2.41 | 4.65E-12 | diacylglycerol kinase iota(DGKI) |
| 2.39 | 1.37E-13 | fibronectin type III domain containing 1(FNDC1) |
| 2.37 | 9.77E-11 | muscle related coiled-coil protein(MURC) |
| 2.37 | 9.52E-10 | carbohydrate sulfotransferase 11(CHST11) |
| 2.37 | 1.84E-11 | solute carrier family 19 member 2(SLC19A2) |
| 2.34 | 4.83E-09 | SLAM family member 8(SLAMF8) |
| 2.34 | 7.53E-15 | transglutaminase 2(TGM2) |
| 2.33 | 1.12E-03 | galanin and GMAP prepropeptide(GAL) |
| 2.33 | 3.93E-10 | NIPA like domain containing 4(NIPAL4) |
| 2.31 | 4.27E-28 | follistatin like 3(FSTL3) |
| 2.29 | 2.32E-16 | RELT tumour necrosis factor receptor(RELT) |
| 2.29 | 6.23E-14 | sterile alpha motif domain containing 11(SAMD11) |
| 2.29 | 1.90E-11 | protocadherin 19(PCDH19) |
| 2.27 | 5.14E-17 | collagen type VII alpha 1 chain(COL7A1) |
| 2.26 | 5.00E-10 | inhibin beta A subunit(INHBA) |
| 2.25 | 1.75E-11 | calbindin 2(CALB2) |
| 2.24 | 7.67E-03 | peptidase inhibitor 16(PI16) |
| 2.24 | 2.84E-04 | C-X-C motif chemokine ligand 5(CXCL5) |
| 2.23 | 3.61E-14 | PC-esterase domain containing 1B(PCED1B) |
| 2.21 | 1.98E-19 | ADAM metallopeptidase domain 19(ADAM19) |
| 2.19 | 4.74E-03 | growth differentiation factor 10(GDF10) |
| 2.19 | 1.46E-07 | leukocyte cell derived chemotaxin 2(LECT2) |
| 2.18 | 9.66E-11 | pleckstrin 2(PLEK2) |
| 2.17 | 1.59E-11 | connective tissue growth factor(CTGF) |
| 2.17 | 4.69E-12 | long intergenic non-protein coding RNA 862(LINC00862) |
| 2.17 | 1.31E-08 | Src-like-adaptor(SLA) |
| 2.15 | 3.21E-19 | SMAD family member 7(SMAD7) |
| 2.15 | 6.16E-22 | protein phosphatase 1 regulatory subunit 13 like(PPP1R13L) |
| 2.12 | 2.96E-09 | collagen type IV alpha 1 chain(COL4A1) |
| 2.11 | 2.80E-22 | paired like homeodomain 2(PITX2) |
| 2.10 | 1.16E-08 | uncharacterized LOC102724849(LOC102724849) |
| 2.09 | 6.58E-17 | spindle apparatus coiled-coil protein 1(SPDL1) |
| 2.09 | 4.17E-06 | growth arrest specific 7(GAS7) |
| 2.09 | 1.41E-08 | C-X-C motif chemokine receptor 5(CXCR5) |
| 2.07 | 1.24E-11 | tensin 1(TNS1) |
| 2.07 | 2.67E-14 | tumour protein p53 inducible protein 3(TP53I3) |
| 2.07 | 2.33E-10 | atypical chemokine receptor 3(ACKR3) |
| 2.06 | 1.71E-21 | sphingosine kinase 1(SPHK1) |
| 2.06 | 9.89E-08 | 5-hydroxytryptamine receptor 2A(HTR2A) |
| 2.06 | 2.71E-06 | endothelial cell specific molecule 1(ESM1) |
| 2.06 | 3.46E-07 | distal-less homeobox 2(DLX2) |
| 2.06 | 1.57E-12 | MICAL C-terminal like(MICALCL) |
| 2.05 | 2.35E-06 | long intergenic non-protein coding RNA 673(LINC00673) |
| 2.04 | 9.18E-10 | stathmin domain containing 1(STMND1) |
| 2.03 | 3.48E-18 | spindle apparatus coiled-coil protein 1(SPDL1) |
| 2.03 | 7.77E-05 | matrix metallopeptidase 10(MMP10) |
| 2.02 | 1.64E-03 | serum amyloid A2(SAA2) |
| 2.02 | 4.91E-04 | uncharacterized LOC389332(LOC389332) |
| 2.02 | 1.29E-11 | snail family transcriptional repressor 1(SNAI1) |
| 2.01 | 2.05E-16 | phosphoribosyl pyrophosphate synthetase 1-like 1(PRPS1L1) |
| -2.00 | 5.30E-04 | polypeptide N-acetylgalactosaminyltransferase 15(GALNT15) |
| -2.01 | 6.22E-03 | R-spondin 3(RSPO3) |
| -2.02 | 3.08E-04 | serpin family B member 2(SERPINB2) |
| -2.02 | 1.63E-09 | chromosome 1 open reading frame 21(C1orf21) |
| -2.04 | 5.06E-10 | NOVA alternative splicing regulator 1(NOVA1) |
| -2.04 | 5.80E-08 | adenosine A2b receptor(ADORA2B) |
| -2.04 | 6.32E-08 | mitochondria localized glutamic acid rich protein(MGARP) |
| -2.05 | 4.04E-11 | caspase 4, apoptosis-related cysteine peptidase pseudogene(LOC643733) |
| -2.08 | 4.04E-11 | ring finger protein 144B(RNF144B) |
| -2.08 | 9.93E-09 | phosphodiesterase 7B(PDE7B) |
| -2.08 | 3.55E-09 | lnc-HRH4-7 |
| -2.08 | 4.36E-10 | transmembrane protein 26(TMEM26) |
| -2.08 | 1.35E-13 | APC down-regulated 1(APCDD1) |
| -2.09 | 1.37E-07 | CNKSR family member 3(CNKSR3) |
| -2.09 | 5.23E-06 | fibroblast growth factor 13(FGF13) |
| -2.10 | 3.06E-15 | guanylate binding protein 2(GBP2) |
| -2.10 | 6.79E-18 | CNKSR family member 3(CNKSR3) |
| -2.11 | 3.71E-13 | LRRN4 C-terminal like(LRRN4CL) |
| -2.11 | 3.04E-11 | ADP ribosylation factor like GTPase 4C(ARL4C) |
| -2.12 | 3.14E-06 | ETS variant 1(ETV1) |
| -2.13 | 4.01E-16 | immunoglobulin superfamily member 10(IGSF10) |
| -2.13 | 8.86E-12 | ecto-NOX disulfide-thiol exchanger 1(ENOX1) |
| -2.13 | 1.11E-11 | interleukin 6 receptor(IL6R) |
| -2.14 | 4.74E-06 | mesoderm specific transcript(MEST) |
| -2.14 | 7.68E-09 | coiled-coil domain containing 170(CCDC170) |
| -2.15 | 4.53E-03 | ankyrin 1(ANK1) |
| -2.16 | 4.09E-13 | interferon induced protein with tetratricopeptide repeats 2(IFIT2) |
| -2.18 | 3.18E-13 | nuclear factor, erythroid 2(NFE2) |
| -2.19 | 1.03E-09 | coiled-coil domain containing 102B(CCDC102B) |
| -2.20 | 7.27E-15 | sprouty RTK signalling antagonist 1(SPRY1) |
| -2.20 | 2.05E-15 | heat shock protein family B (small) member 3(HSPB3) |
| -2.21 | 3.55E-05 | doublecortin like kinase 1(DCLK1) |
| -2.21 | 2.48E-14 | toll like receptor 3(TLR3) |
| -2.21 | 6.69E-08 | monoamine oxidase A(MAOA) |
| -2.22 | 5.05E-08 | caspase 1(CASP1) |
| -2.22 | 1.21E-04 | fibronectin leucine rich transmembrane protein 3(FLRT3) |
| -2.24 | 5.91E-12 | purinergic receptor P2X 7(P2RX7) |
| -2.24 | 9.00E-05 | receptor interacting serine/threonine kinase 4(RIPK4) |
| -2.26 | 2.27E-10 | breast carcinoma amplified sequence 1(BCAS1) |
| -2.26 | 1.95E-10 | chromosome 1 open reading frame 167(C1orf167) |
| -2.26 | 3.80E-18 | regulator of calcineurin 2(RCAN2) |
| -2.27 | 1.74E-05 | serum deprivation response(SDPR) |
| -2.27 | 6.32E-07 | angiomotin(AMOT) |
| -2.29 | 2.29E-17 | transmembrane and tetratricopeptide repeat containing 1(TMTC1) |
| -2.30 | 2.25E-07 | roundabout guidance receptor 2(ROBO2) |
| -2.31 | 1.72E-12 | acyl-CoA synthetase short-chain family member 1(ACSS1) |
| -2.31 | 1.85E-08 | kinesin family member 5C(KIF5C) |
| -2.32 | 2.17E-07 | potassium voltage-gated channel subfamily B member 1(KCNB1) |
| -2.32 | 2.29E-08 | atypical chemokine receptor 4(ACKR4) |
| -2.33 | 1.08E-15 | long intergenic non-protein coding RNA 341(LINC00341) |
| -2.33 | 1.87E-08 | cathepsin C(CTSC) |
| -2.33 | 7.86E-11 | coiled-coil domain containing 102B(CCDC102B) |
| -2.35 | 5.32E-07 | annexin A3(ANXA3) |
| -2.35 | 2.40E-14 | cadherin related family member 3(CDHR3) |
| -2.37 | 9.55E-15 | GTPase, IMAP family member 2(GIMAP2) |
| -2.37 | 1.09E-06 | potassium voltage-gated channel subfamily J member 2(KCNJ2) |
| -2.38 | 2.17E-15 | armadillo repeat containing 4(ARMC4) |
| -2.38 | 6.38E-12 | lnc-SMC1B-2 |
| -2.39 | 1.11E-10 | uncharacterized LOC101928188(LOC101928188) |
| -2.40 | 8.03E-15 | death associated protein kinase 1(DAPK1) |
| -2.40 | 4.70E-22 | odd-skipped related transciption factor 2(OSR2) |
| -2.42 | 3.08E-09 | uncharacterized LOC101929174(LOC101929174) |
| -2.43 | 5.07E-08 | uncharacterized LOC101929641(LOC101929641) |
| -2.43 | 5.93E-04 | family with sequence similarity 65 member B(FAM65B) |
| -2.43 | 9.38E-07 | nuclear receptor subfamily 0 group B member 1(NR0B1) |
| -2.43 | 6.58E-09 | ecotropic viral integration site 2A(EVI2A) |
| -2.45 | 1.69E-10 | uncharacterized LOC101929174(LOC101929174) |
| -2.45 | 2.32E-16 | solute carrier family 9 member A9(SLC9A9) |
| -2.46 | 4.43E-22 | TBC1 domain family member 8(TBC1D8) |
| -2.49 | 3.75E-07 | calcium voltage-gated channel auxiliary subunit beta 2(CACNB2) |
| -2.49 | 2.24E-03 | ankyrin 1(ANK1) |
| -2.49 | 5.62E-10 | reticulon 4 receptor like 1(RTN4RL1) |
| -2.50 | 3.19E-08 | microtubule associated protein tau(MAPT) |
| -2.51 | 2.91E-07 | mesenchyme homeobox 2(MEOX2) |
| -2.56 | 7.53E-15 | secreted and transmembrane 1(SECTM1) |
| -2.57 | 1.08E-05 | myelin basic protein(MBP) |
| -2.58 | 4.86E-10 | long intergenic non-protein coding RNA 908(LINC00908) |
| -2.58 | 1.17E-16 | potassium voltage-gated channel modifier subfamily S member 2(KCNS2) |
| -2.58 | 9.89E-11 | ST8 alpha-N-acetyl-neuraminide alpha-2,8-sialyltransferase 1(ST8SIA1) |
| -2.62 | 6.27E-11 | growth differentiation factor 5(GDF5) |
| -2.63 | 1.17E-16 | solute carrier family 7 member 14(SLC7A14) |
| -2.65 | 2.70E-09 | myosin VIIA and Rab interacting protein(MYRIP) |
| -2.68 | 1.60E-13 | signal induced proliferation associated 1 like 2(SIPA1L2) |
| -2.71 | 6.83E-11 | keratin associated protein 1-5(KRTAP1-5) |
| -2.73 | 2.95E-07 | KIAA1217(KIAA1217) |
| -2.75 | 8.12E-20 | RAB7B, member RAS oncogene family(RAB7B) |
| -2.77 | 8.87E-17 | RAS like family 11 member A(RASL11A) |
| -2.78 | 2.63E-26 | fatty acid hydroxylase domain containing 2(FAXDC2) |
| -2.89 | 1.63E-11 | ankyrin repeat domain 33B(ANKRD33B) |
| -2.92 | 8.50E-14 | pentraxin 3(PTX3) |
| -2.92 | 2.82E-05 | cadherin 18(CDH18) |
| -2.92 | 5.40E-09 | integrin subunit beta 8(ITGB8) |
| -2.93 | 1.03E-11 | transmembrane protein 26(TMEM35) |
| -2.98 | 6.44E-06 | chromosome 10 open reading frame 105(C10orf105) |
| -3.06 | 2.97E-13 | keratin associated protein 1-5(KRTAP1-5) |
| -3.09 | 7.54E-11 | semaphorin 3A(SEMA3A) |
| -3.09 | 2.47E-16 | long intergenic non-protein coding RNA 908(LINC00908) |
| -3.10 | 4.30E-16 | solute carrier family 7 member 14(SLC7A14) |
| -3.15 | 1.75E-11 | interferon induced protein with tetratricopeptide repeats 1(IFIT1) |
| -3.22 | 2.37E-06 | lipid phosphate phosphatase-related 4(LPPR4) |
| -3.27 | 1.21E-15 | sterile alpha motif domain containing 12(SAMD12) |
| -3.47 | 1.66E-11 | alcohol dehydrogenase 1C (class I), gamma polypeptide(ADH1C) |
| -3.59 | 4.33E-12 | alcohol dehydrogenase 1A (class I), alpha polypeptide(ADH1A) |
| -3.68 | 3.13E-14 | peroxisome proliferator activated receptor gamma(PPARG) |
| -3.70 | 5.72E-11 | chromosome 11 open reading frame 87(C11orf87) |
| -4.17 | 3.06E-15 | periplakin(PPL) |
| -4.75 | 3.10E-18 | KIT proto-oncogene receptor tyrosine kinase(KIT) |

**Supplementary Table 5.**

| **Non-treated** | | |  | **TGF-β1 treated** | | |
| --- | --- | --- | --- | --- | --- | --- |
| **LogFC** | **Adj. p value** | **Gene name (ID)** |  | **LogFC** | **Adj. p value** | **Gene name (ID)** |
| 5.63 | 2.57E-04 | IGF1 |  | 4.05 | 1.23E-02 | IGF1 |
| 5.18 | 4.65E-04 | IGF1 |  | 4.02 | 3.75E-02 | IGF1 |
| -2.51 | 1.21E-03 | WNT4 |  | 3.18 | 4.52E-03 | CXCL1 |
| -3.70 | 2.67E-04 | ASPN |  | 2.85 | 5.51E-03 | CXCL1 |
| -3.79 | 4.03E-04 | ASPN |  | -2.79 | 1.86E-03 | WNT4 |
| -5.06 | 4.45E-06 | WNT4 |  | -3.08 | 1.36E-02 | ASPN |
|  |  |  |  | -3.24 | 1.25E-02 | ASPN |
|  |  |  |  | -3.69 | 2.49E-02 | WNT4 |
