# Supplementary figures and images for: Differential Expression of Insulin-Like Growth Factor 1 and Wnt Family Member 4 Correlates With Functional Heterogeneity of Human Dermal Fibroblasts
Source: Front Cell Dev Biol. 2021 Apr 6;9:628039. doi: 10.3389/fcell.2021.628039 (PMC8056032; doi:10.3389/fcell.2021.628039)

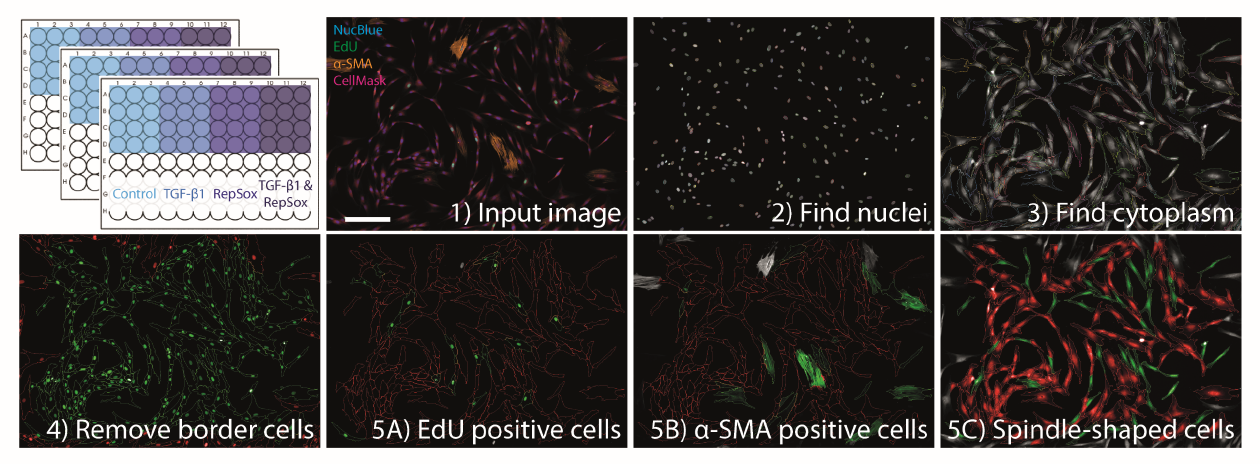

Supplement: Supplementary file 2 [file Image_1.tif]

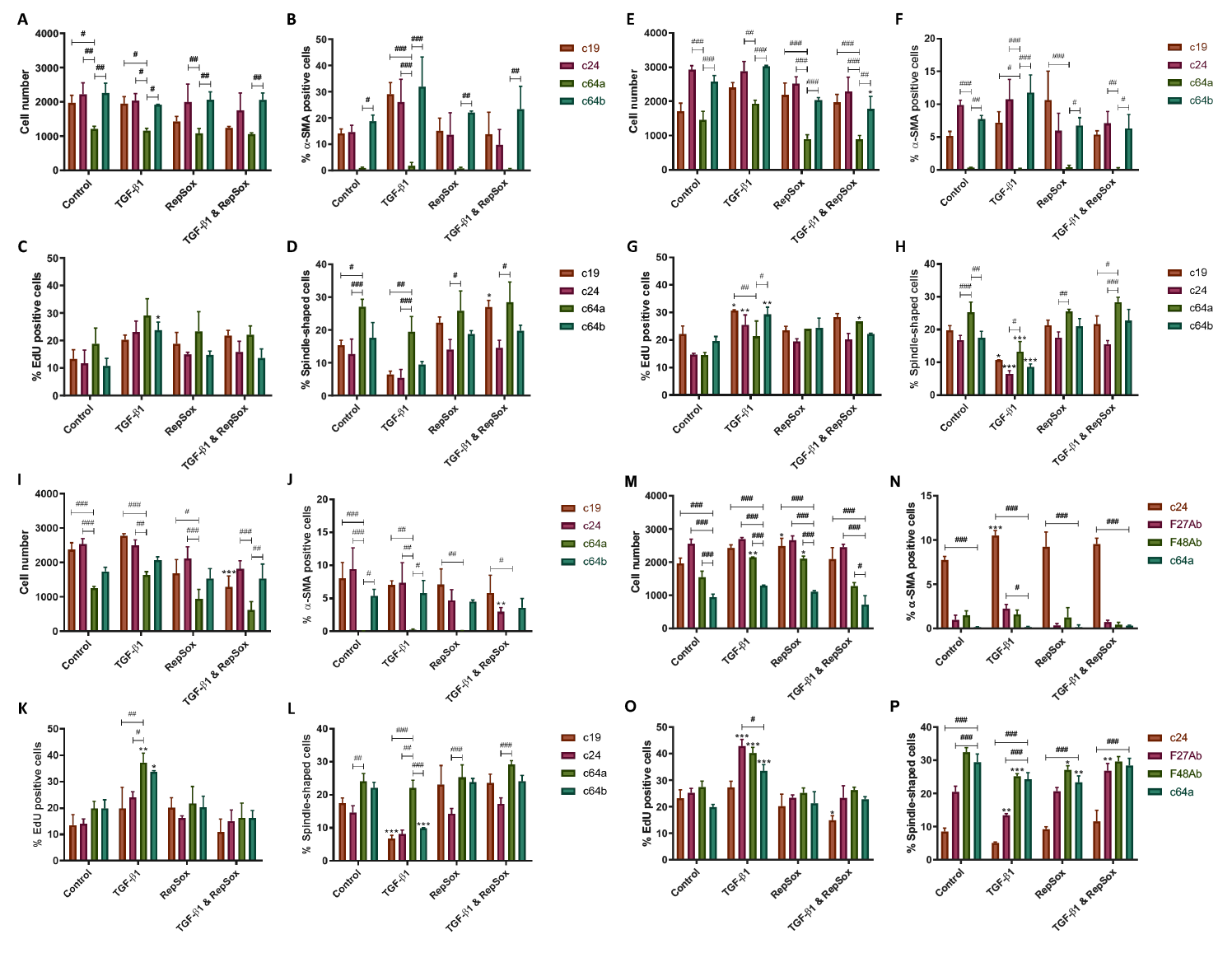

Supplement: Supplementary file 3 [file Image_2.tif]

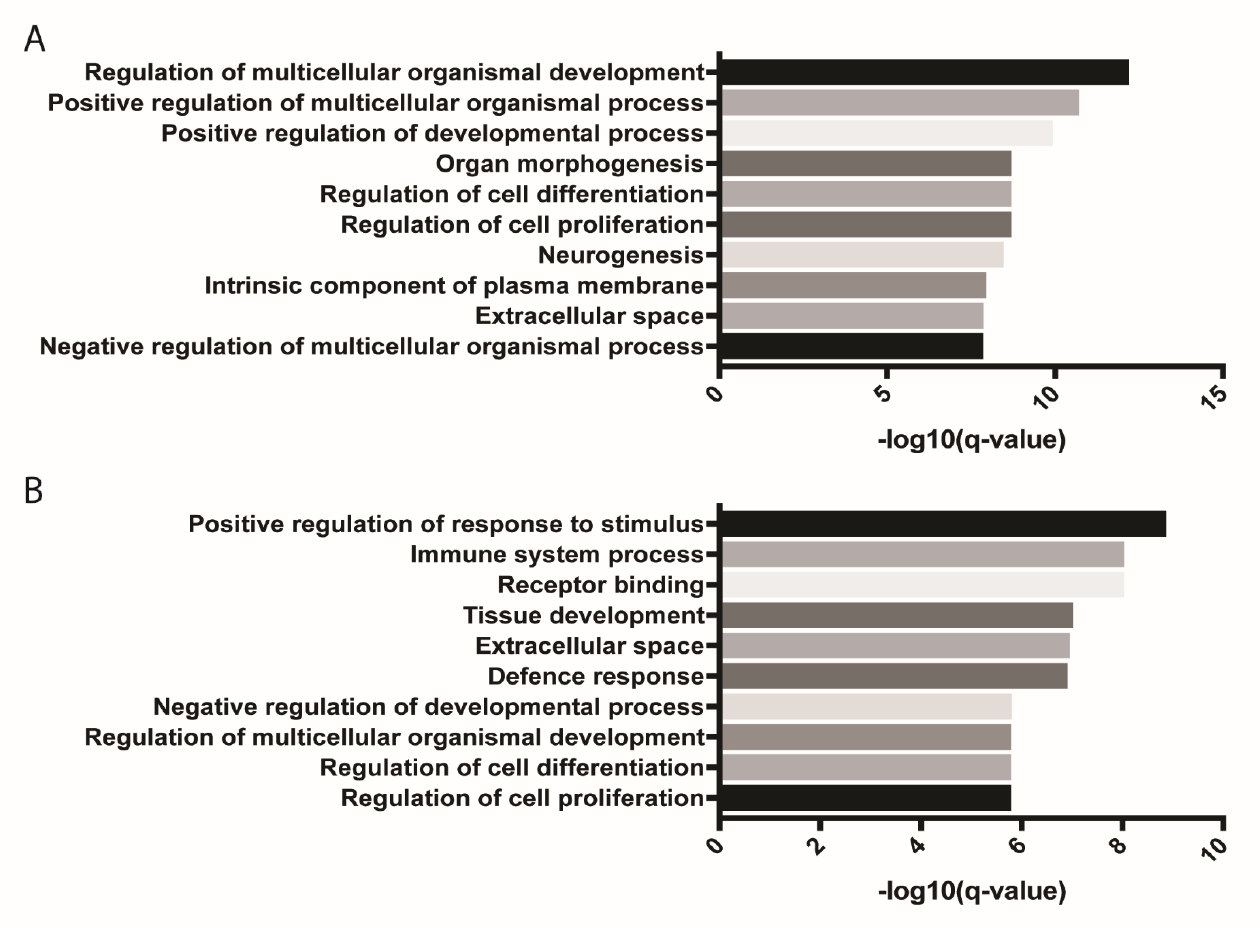

Supplement: Supplementary file 4 [file Image_3.tif]

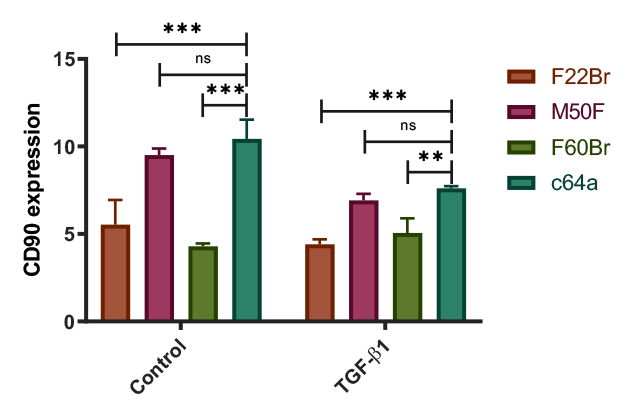

Supplement: Supplementary file 5 [file Image_4.tif]

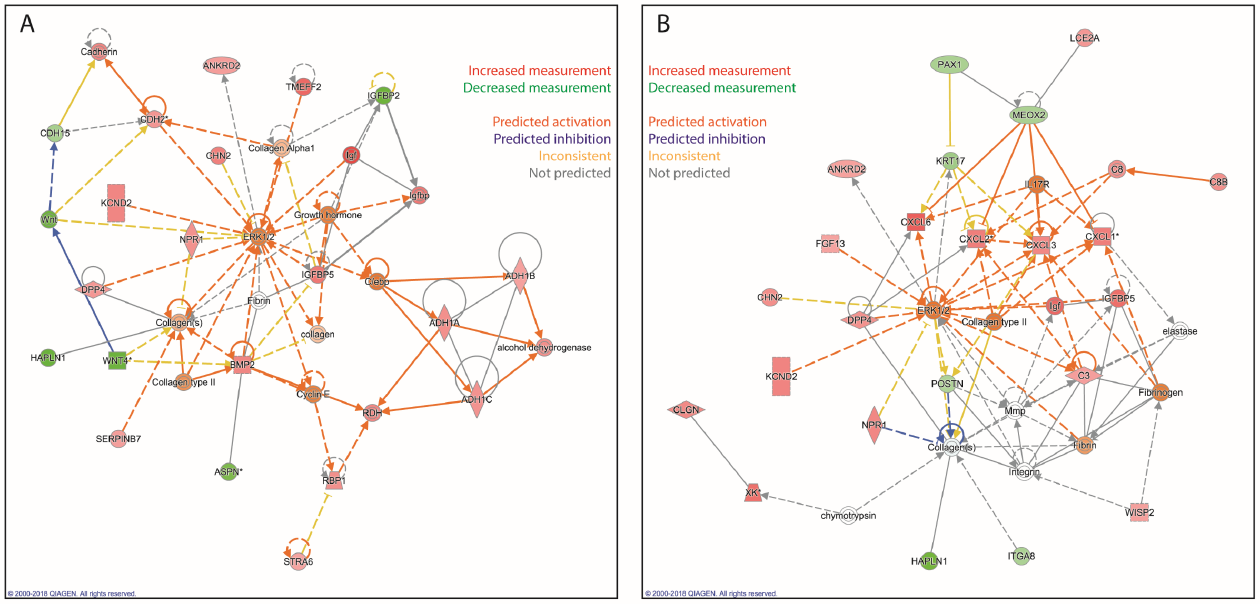

Supplement: Supplementary file 6 [file Image_5.tif]

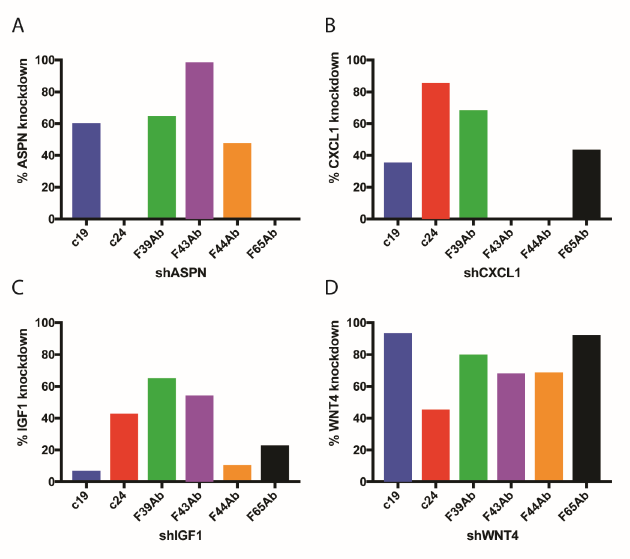

Supplement: Supplementary file 7 [file Image_6.tif]

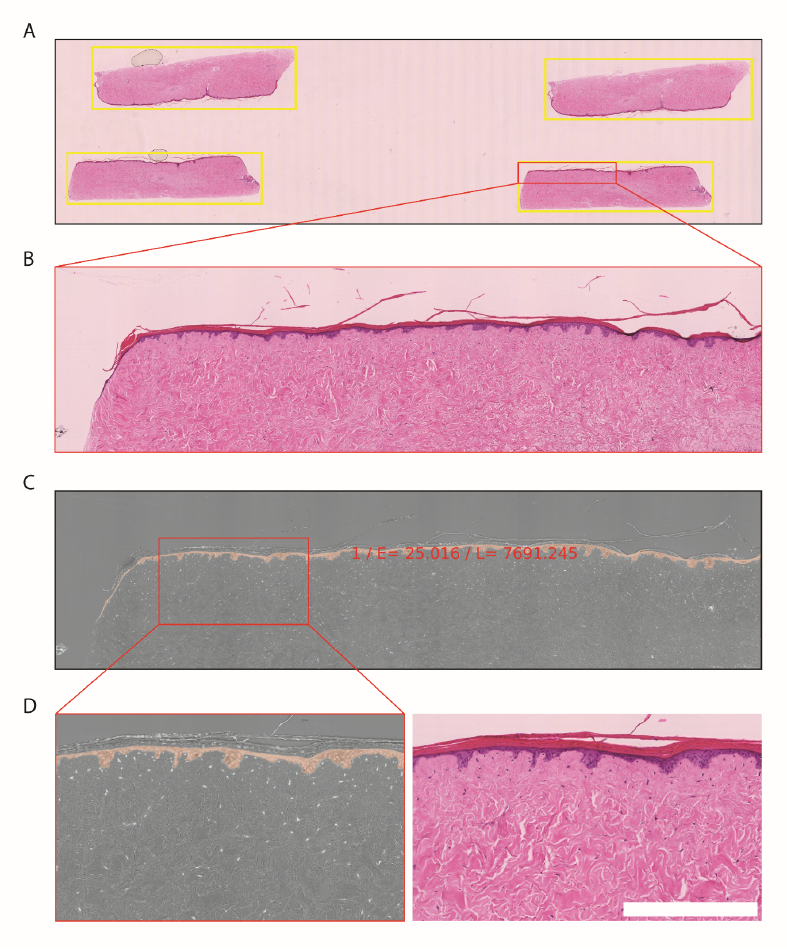

Supplement: Supplementary file 9 [file Image_8.tif]
